# Supplementary figures and images for: Integrative QTL analysis of gene expression and chromatin accessibility identifies multi-tissue patterns of genetic regulation
Source: PLoS Genet. 2020 Jan 21;16(1):e1008537. doi: 10.1371/journal.pgen.1008537 (PMC7010298; doi:10.1371/journal.pgen.1008537)

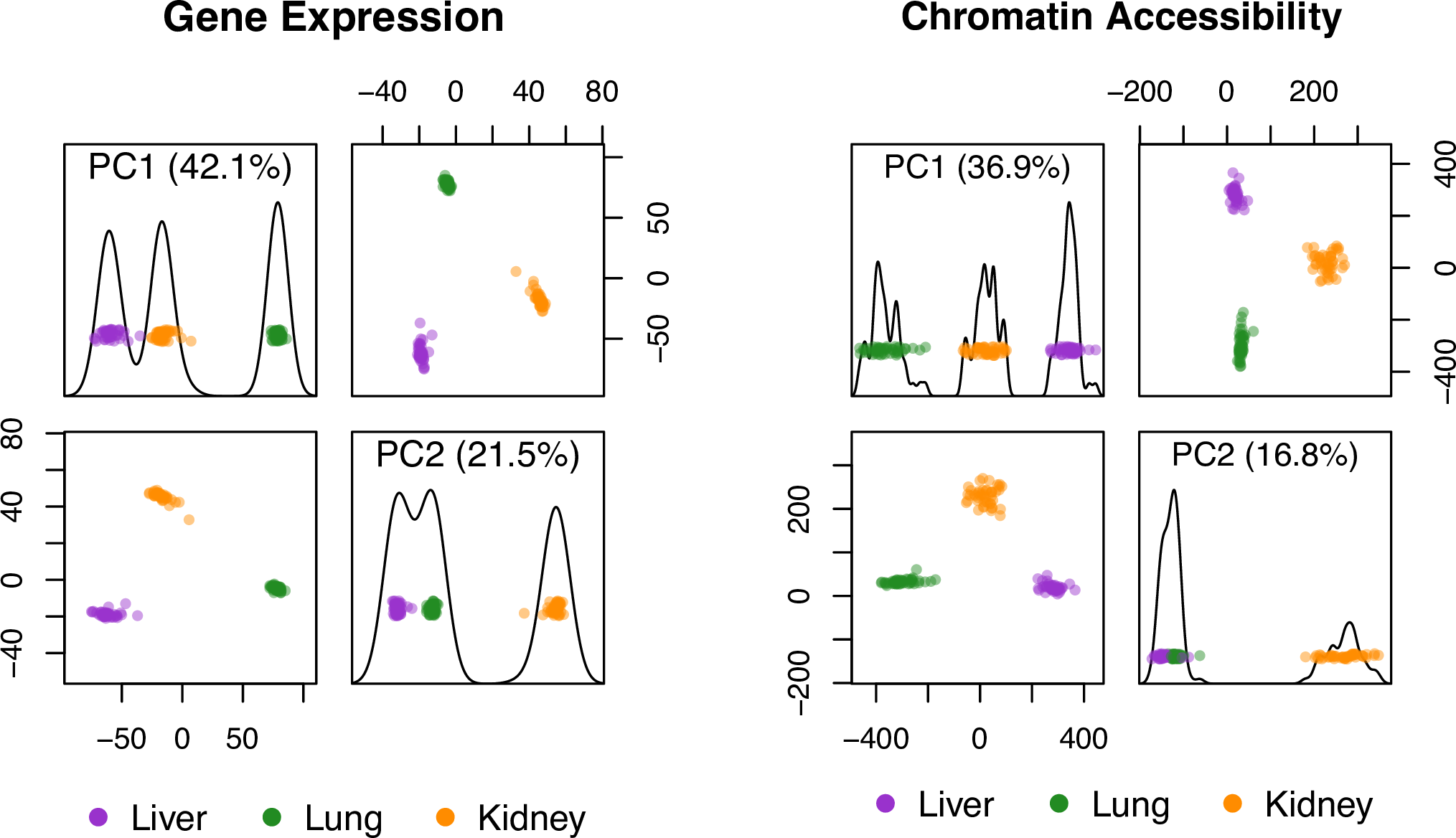

Supplement: S1 Fig — Molecular traits for liver (purple), lung (green), and kidney (orange) tissue samples were derived from RNA-seq and ATAC-seq data. Principal components (PC) 1 and 2 capture a majority of the variation and show a greater amount of between tissue variability than within tissue variability. (TIF) [file pgen.1008537.s001.tif]

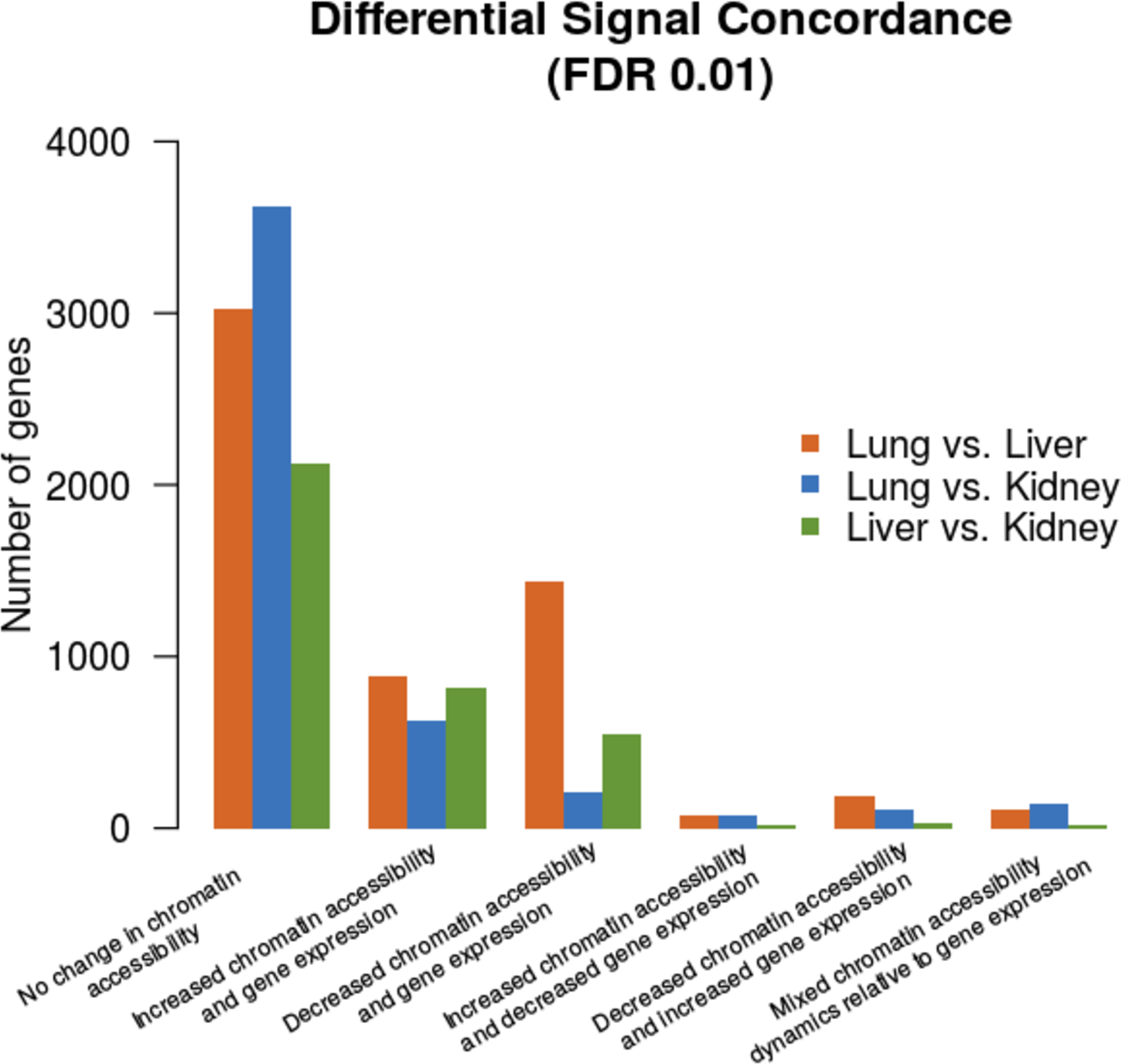

Supplement: S2 Fig — Genes were categorized by the direction of the difference in expression and chromatin accessibility in their promoter regions. (TIF) [file pgen.1008537.s002.tif]

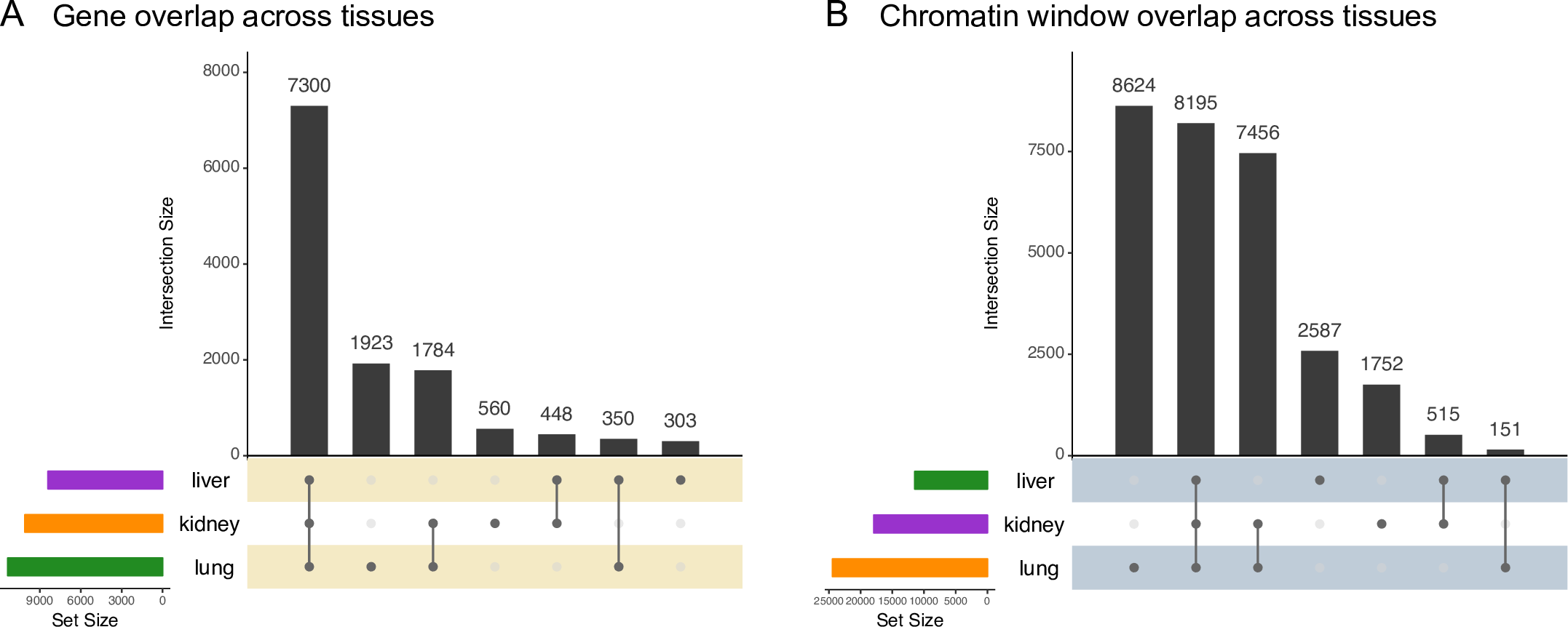

Supplement: S3 Fig — Sequence traits were filtered to remove outcomes more likely to cause spurious QTL signals. Genes with TPM ≤ 1 and chromatin windows with TMP ≤ 5 for ≥ 50% of samples were removed from analysis. After this filtering process, lung had the greatest number of traits analyzed, for both genes and chromatin windows, followed by kidney and then liver. (TIF) [file pgen.1008537.s003.tif]

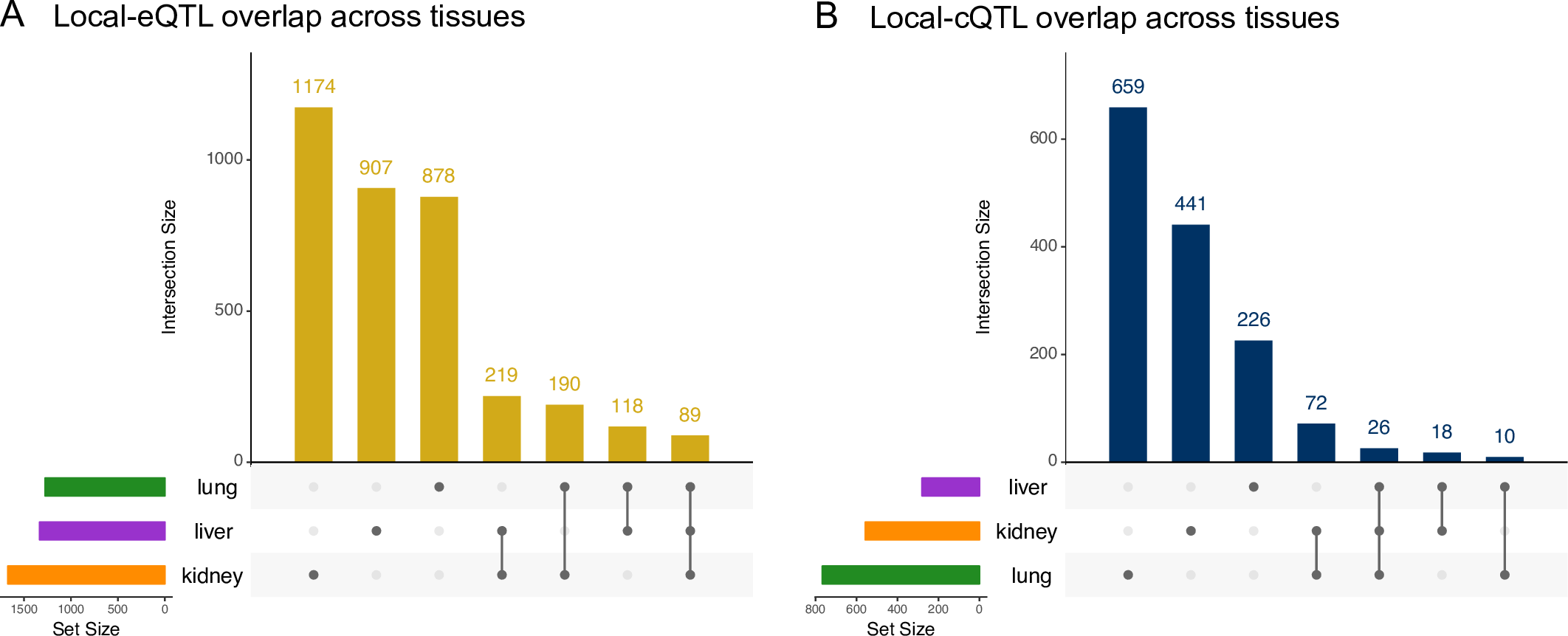

Supplement: S4 Fig — The majority of sequence traits with a local-QTL detected were identified in only a single tissue. Kidney had the highest number of local-eQTL, whereas lung had the highest number of local-cQTL. Liver had a relative lack of local-cQTL, which may relate to its having the fewest chromatin windows analyzed (S3B Fig). Results included local-QTL detected with Analysis G (FDR ≤ 0.1), Analysis C (FDR ≤ 0.1), and Analysis L (genome-wide and chromosome-wide). (TIF) [file pgen.1008537.s004.tif]

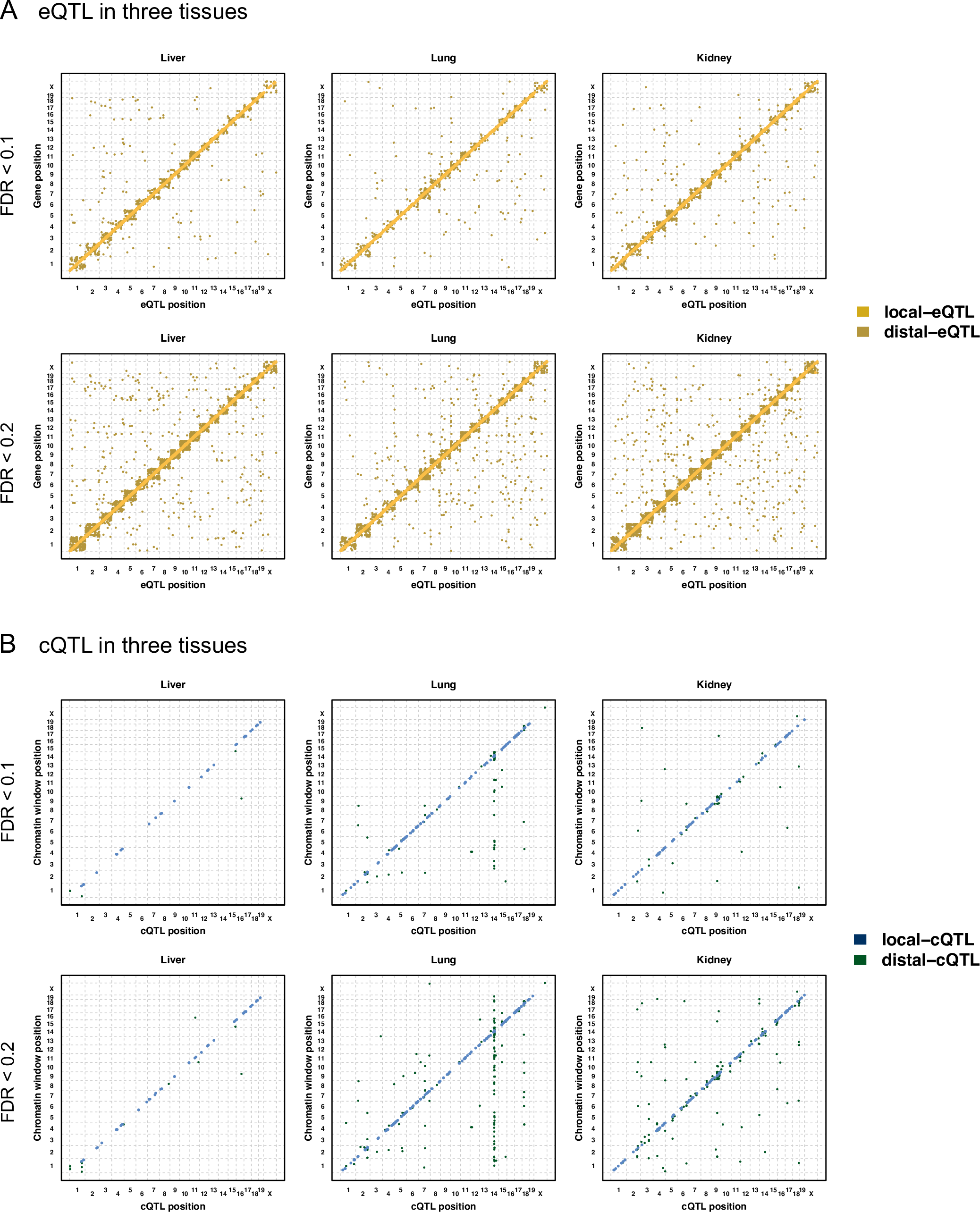

Supplement: S5 Fig — QTL map plots of (A) eQTL and (B) cQTL with FDR controlled at 0.1 and 0.2 for liver, lung, and kidney. Detected QTL from Analysis G (multi-stage FDR) and Analysis C (chromosome-wide FDR) are included. Analysis C, which uses FDR control for chromosome-wide significant QTL, produces a large number of intra-chromosomal distal-QTL. The y-axis represents the genomic position of the gene or chromatin site, and the x-axis represents the genomic position of the QTL. Local-QTL appear as dots along the diagonal. (TIF) [file pgen.1008537.s005.tif]

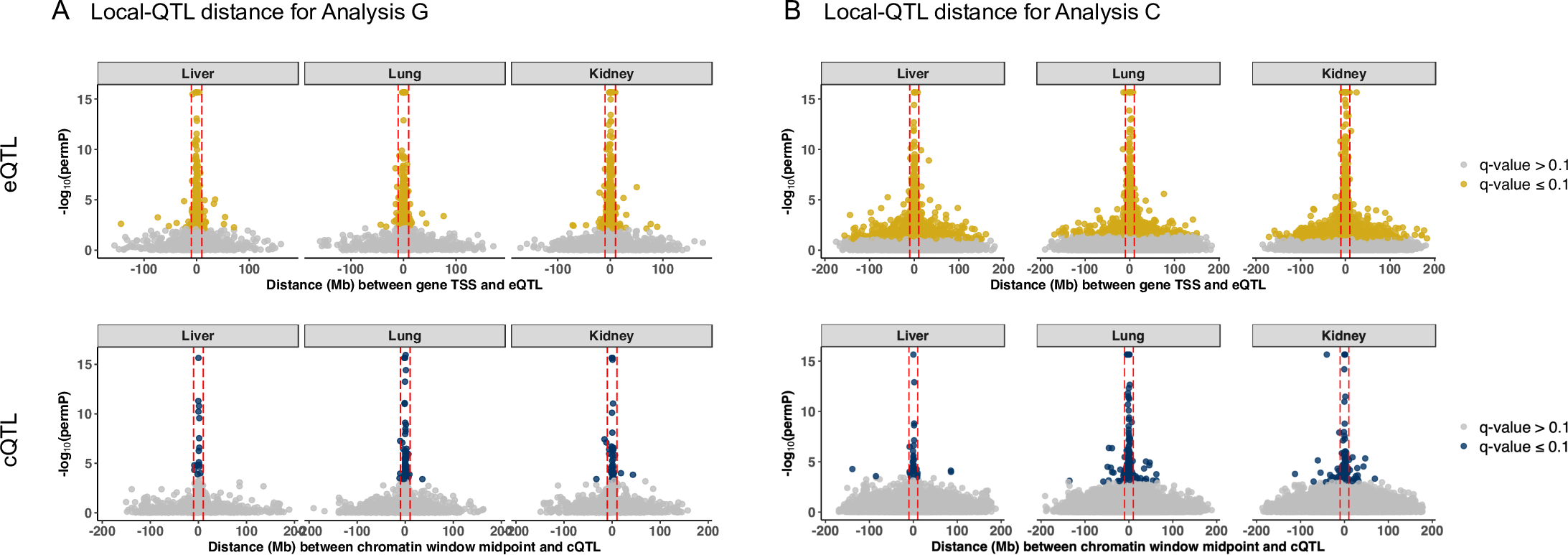

Supplement: S6 Fig — The permutation-based p-value (permP) from (A) Analysis G and (B) Analysis C for eQTL and cQTL by their distance (Mb) from the gene TSS and the midpoint of the chromatin site. Inter-chromosomal distal-QTL are not included. The red dashed lines represent ±10Mb of the gene TSS or the midpoint of the chromatin site for classifying QTL as local or distal. Significant signals (yellow or blue), based on FDR ≤ 0.1, are largely local. Analysis C detects many more intra-chromosomal distal-QTL. (TIF) [file pgen.1008537.s006.tif]

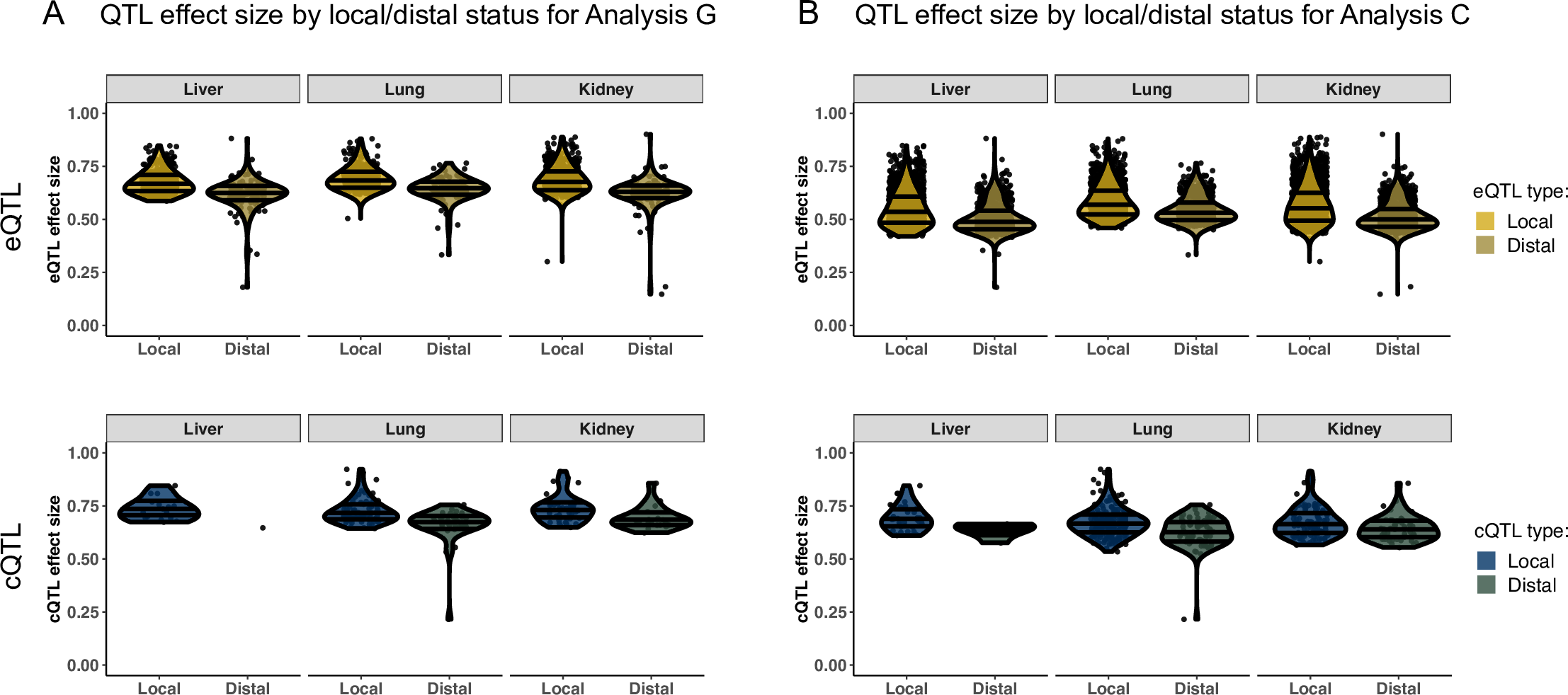

Supplement: S7 Fig — Each dot represents a QTL detected through either (A) Analysis G or (B) Analysis C with FDR ≤ 0.1. The three horizontal bars represent the 25th, 50th, and 75th quantiles of QTL effect sizes for all local-QTL per tissue. More local-eQTL are detected and have higher effects than distal-QTL. Analysis C detects a large number of intra-chromosomal distal-QTL that Analysis G does not, many of which have low effect sizes. Effect size estimates are based on a fixed effects model. (TIF) [file pgen.1008537.s007.tif]

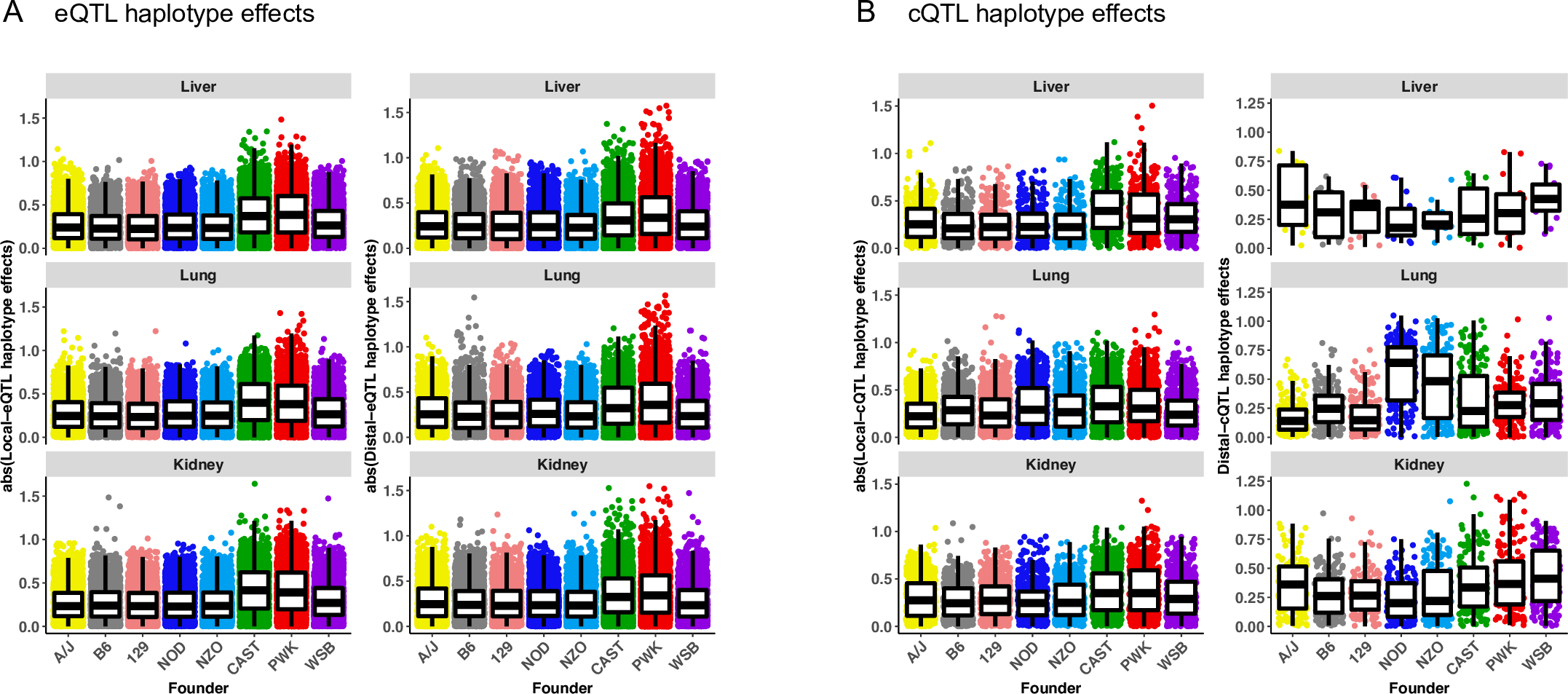

Supplement: S8 Fig — Haplotype effects were estimated as BLUPs, which are constrained and centered around 0. Each QTL is represented by an 8-element effect vector. Founders with more extreme effects are identified by comparing the absolute values of effects. Founder haploytpe effect trends for eQTL are similar to cQTL. The trends are unstable in distal-cQTL because so few are identified. (TIF) [file pgen.1008537.s008.tif]

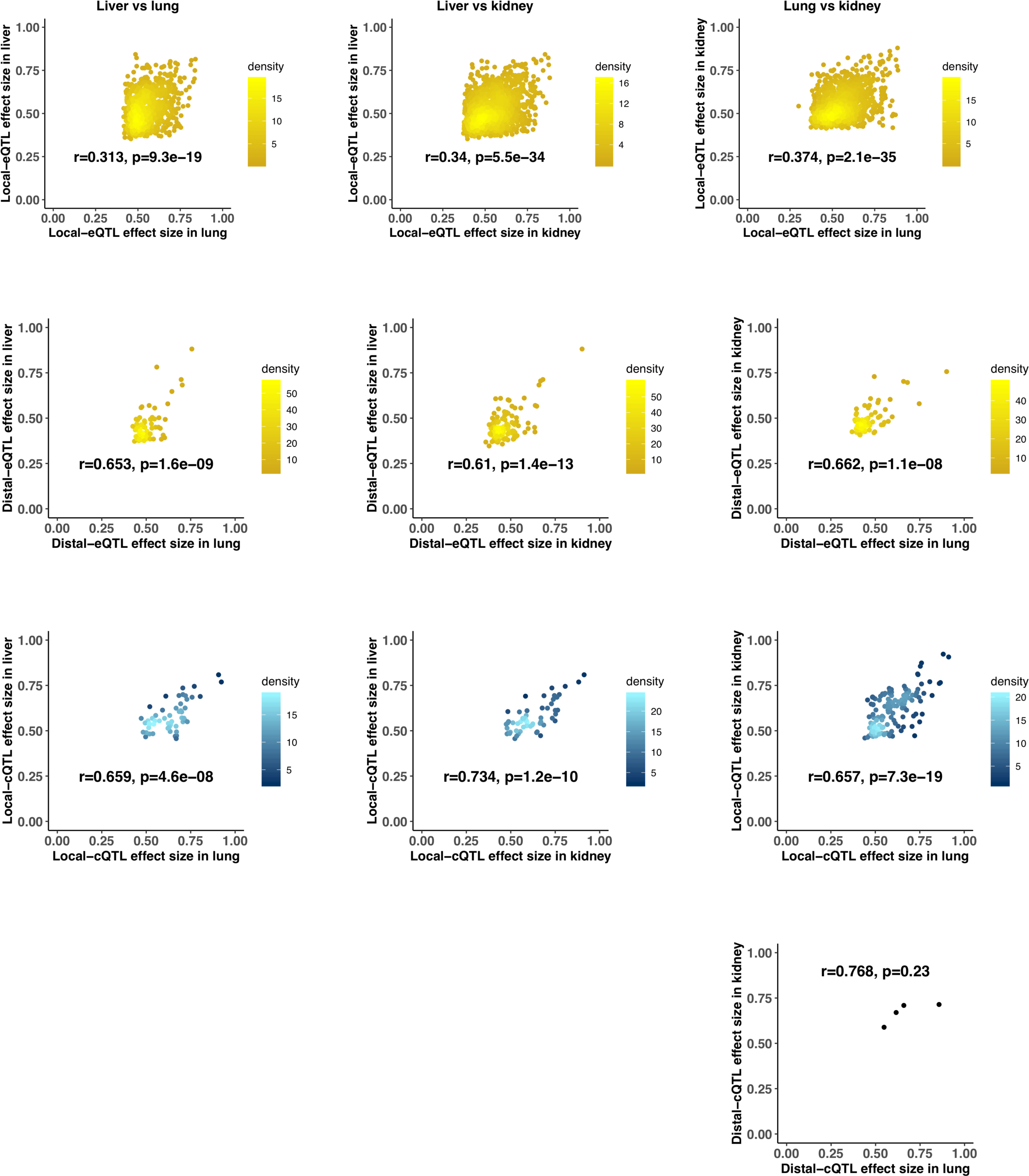

Supplement: S9 Fig — Comparisons of QTL effects sizes between (liver/lung) are in the left column, (liver/kidney) middle column, and (lung/kidney) right column. eQTL are yellow and cQTL are blue. Local-eQTL are plotted in the top row, distal-eQTL in the second row, local-cQTL in the third row, and distal-cQTL in the bottom row, with only four pairs detected in (lung/kidney). (TIF) [file pgen.1008537.s009.tif]

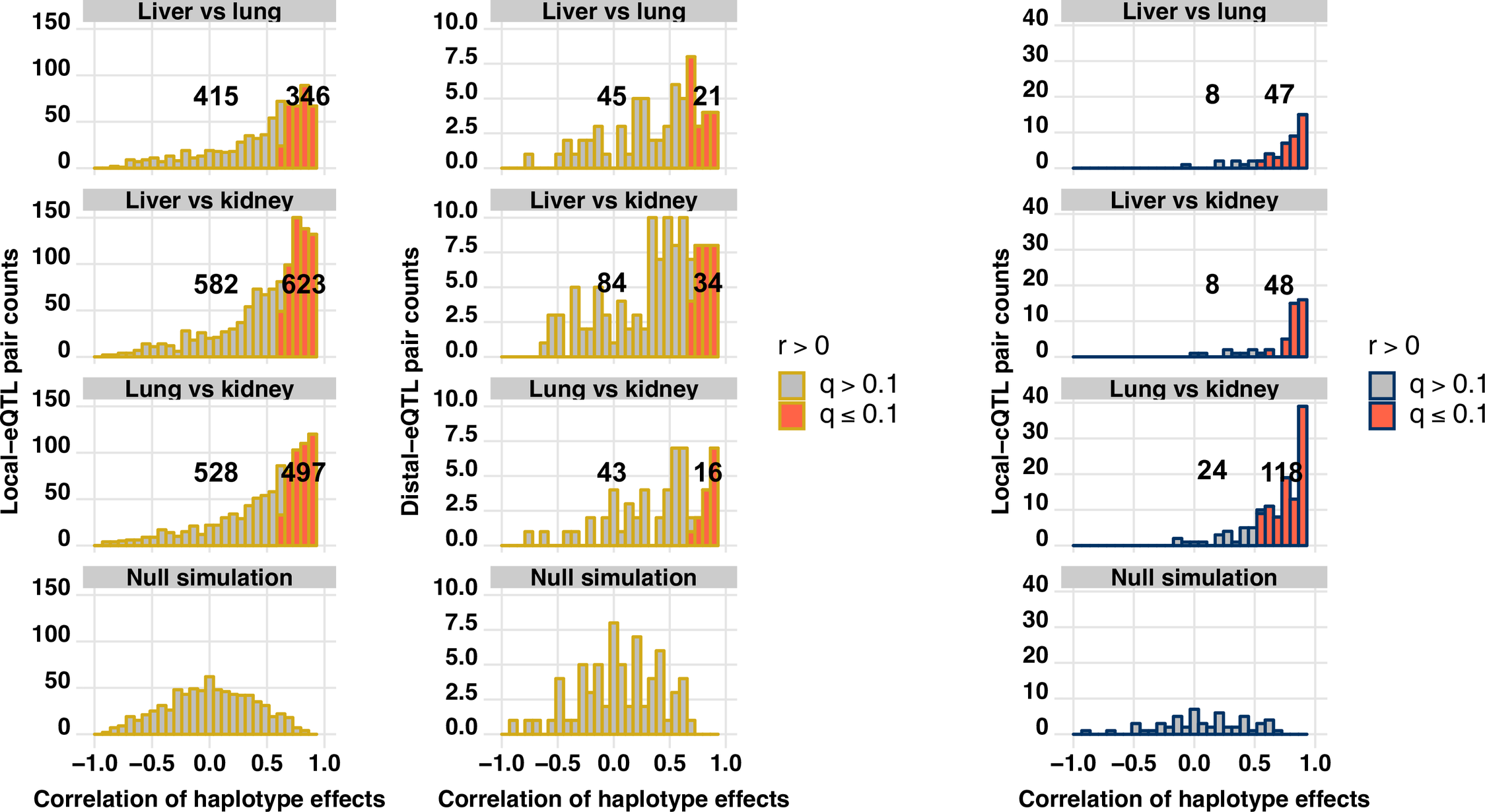

Supplement: S10 Fig — There was an excess of significant positively correlated haplotype effects in QTL pairs across tissues for gene expression and chromatin accessibility. Pairs of QTL observed in multiple tissues were defined for local-eQTL (left column), distal-eQTL (middle column), and local-cQTL (right column). Only four pairs of distal-cQTL were observed, all shared between lung and kidney. A right-tailed test the correlation between haplotype effects (HA: r > 0) was performed for each QTL pair, producing p-values that were then FDR adjusted. Null simulations of uncorrelated 8-element vector pairs for each class of QTL and pairwise tissue comparison emphasize the observed enrichment in correlated haplotype effects between QTL pairs. (TIF) [file pgen.1008537.s010.tif]

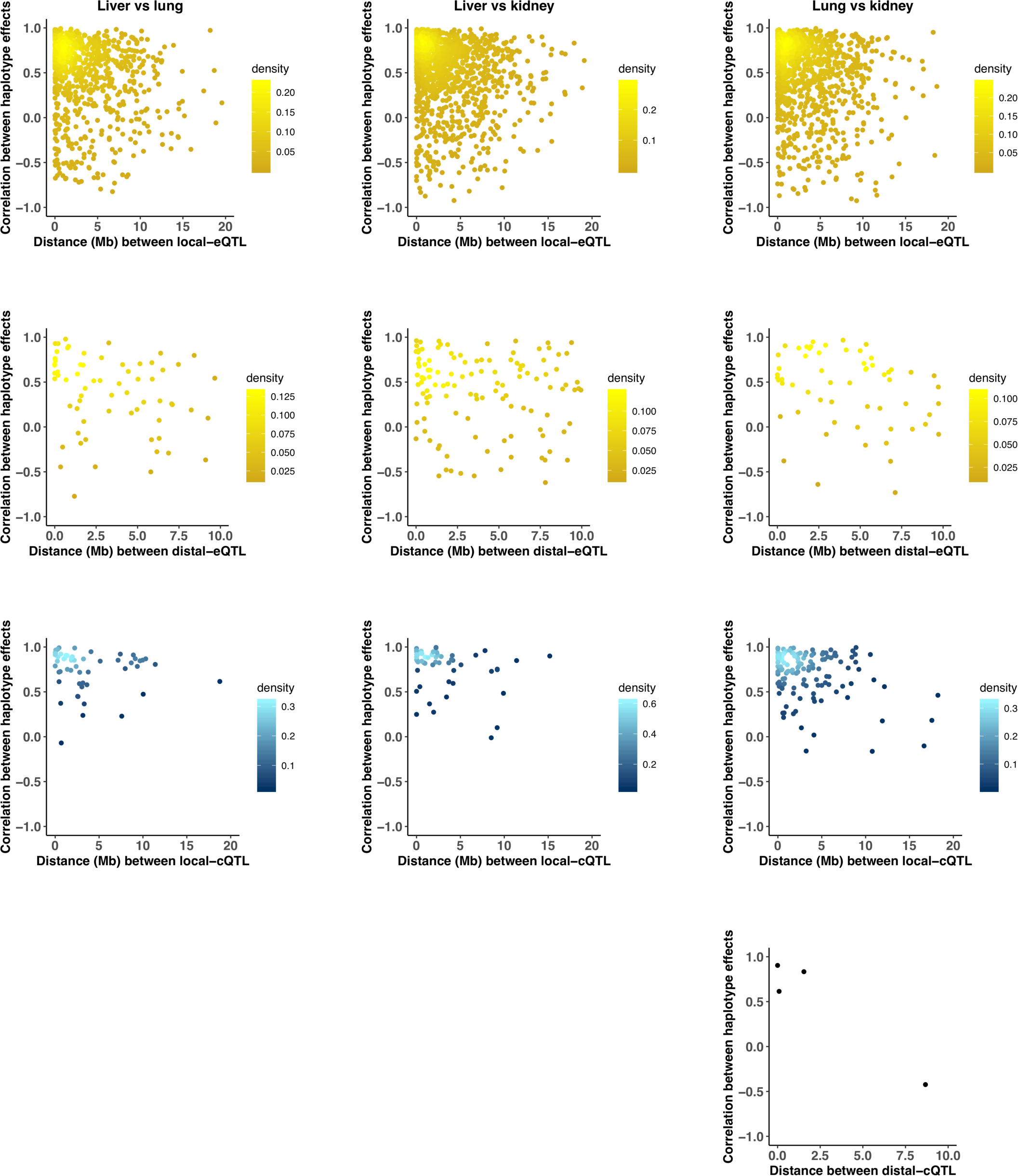

Supplement: S11 Fig — Haplotype effects were estimated as constrained BLUPs. Pairwise correlations of the 8-element effect vectors were calculated for QTL pairs, and plotted again the distance between the QTL coordinates in Mb for (liver/lung) in the left column, (liver/kidney) in the middle column, and (lung/kidney) in the right column. Single eQTL and cQTL pairs are represented as a yellow and blue dots, respectively. Local-eQTL are shown in the top row, distal-eQTL in the second row, local-cQTL in the third row, and distal c-QTL in the bottom row, for which only four pairs were detected in (lung/kidney). (TIF) [file pgen.1008537.s011.tif]

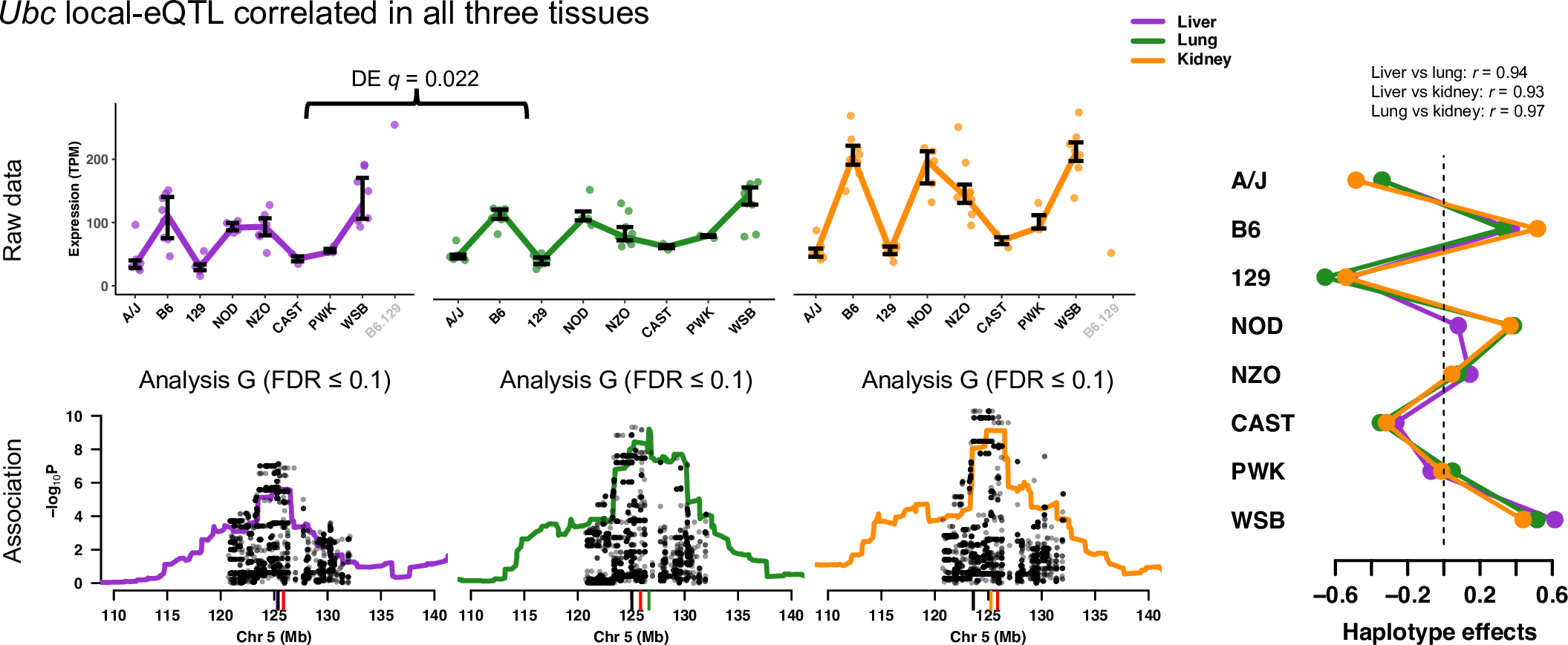

Supplement: S12 Fig — The local-eQTL consistently drove higher expression when the B6, NOD, NZO, and WSB haploytpes were present. Expression levels in liver and lung were found to be significantly different (q = 0.022). The estimated haplotype effects were highly consistent with the expression data, represented as interquartile bars categorized by most likely diplotype. The haplotype and variant associations in the eQTL regions were similar across tissues, suggesting they may represent the same causal origin. The red tick represents the Ubc TSS, the black tick represents the peak variant association, and the colored ticks represent the peak haplotype association for each tissue. (TIF) [file pgen.1008537.s012.tif]

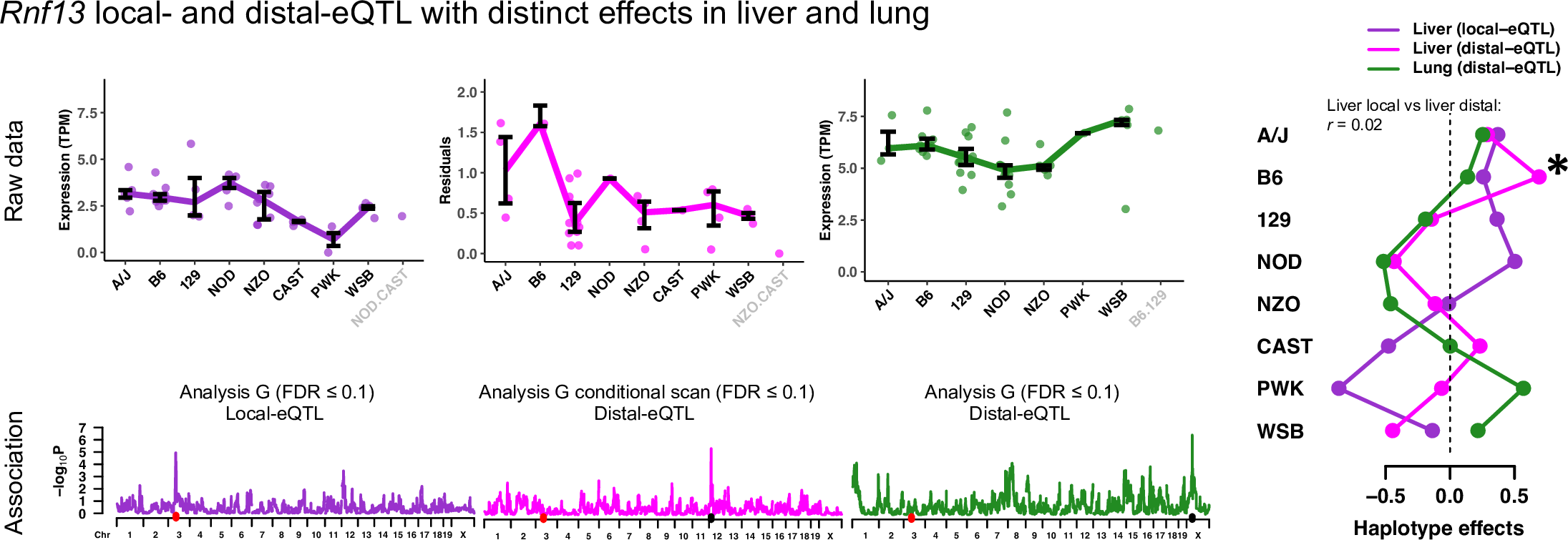

Supplement: S13 Fig — A strong local-eQTL was detected in liver, and after conditioning on it, a statistically significant distal-eQTL was detected (Analysis G) on chromosome 12, largely driven by the B6 haplotype, distinct from the local-eQTL. The unique haplotype effect patterns for each eQTL can be seen in both the expression data, represented by interquartile bars for most likely diplotype, and the estimated effects. The red tick marks the Rnf13 TSS and the black tick marks the location of distal-eQTL. Another strong distal-eQTL was detected on the X chromosome in lung. (TIF) [file pgen.1008537.s013.tif]

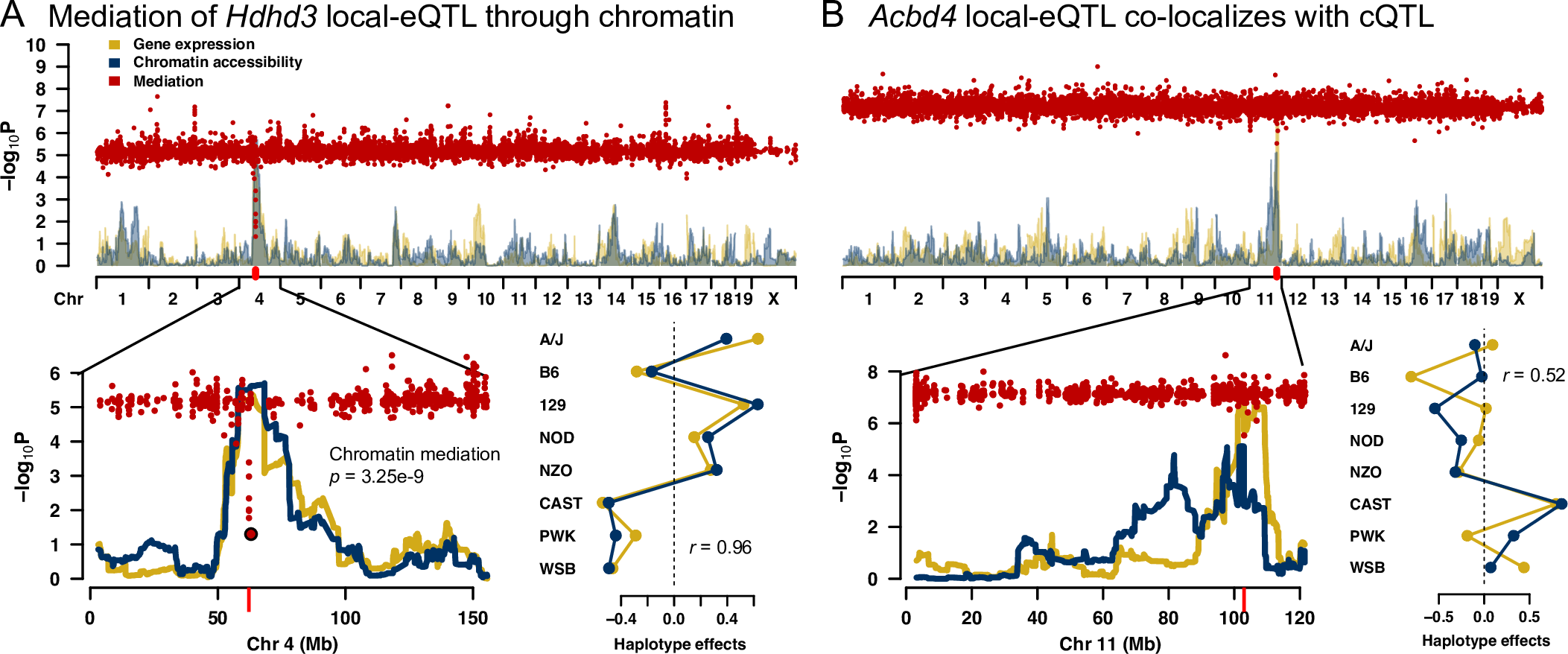

Supplement: S14 Fig — The approach used to detect mediation through chromatin accessibility requires that the eQTL and cQTL co-localize (both within 10Mb of the gene TSS), as well as possess similar haplotype effect patterns. Co-localizing cQTL are observed for local-eQTL for both (A) Hdhd3 in liver and (B) Acbd4 in kidney. QTL and mediation scans are shown, with chromosomes 4 and 11 blown up for Hdhd3 and Acbd4, respectively. The red ticks denote the TSS for both genes. The haplotype effects for the eQTL and cQTL are highly correlated (r = 0.96) for Hdhd3, but not for Acbd4 (r = 0.55). Strong mediation of the Hdhd3 eQTL through chromatin is detected, but not for Acbd4. The effect size of the co-localizing cQTL to Acbd4 is smaller than its eQTL, also inconsistent with the relationship depicted in Fig 6A. (TIF) [file pgen.1008537.s014.tif]

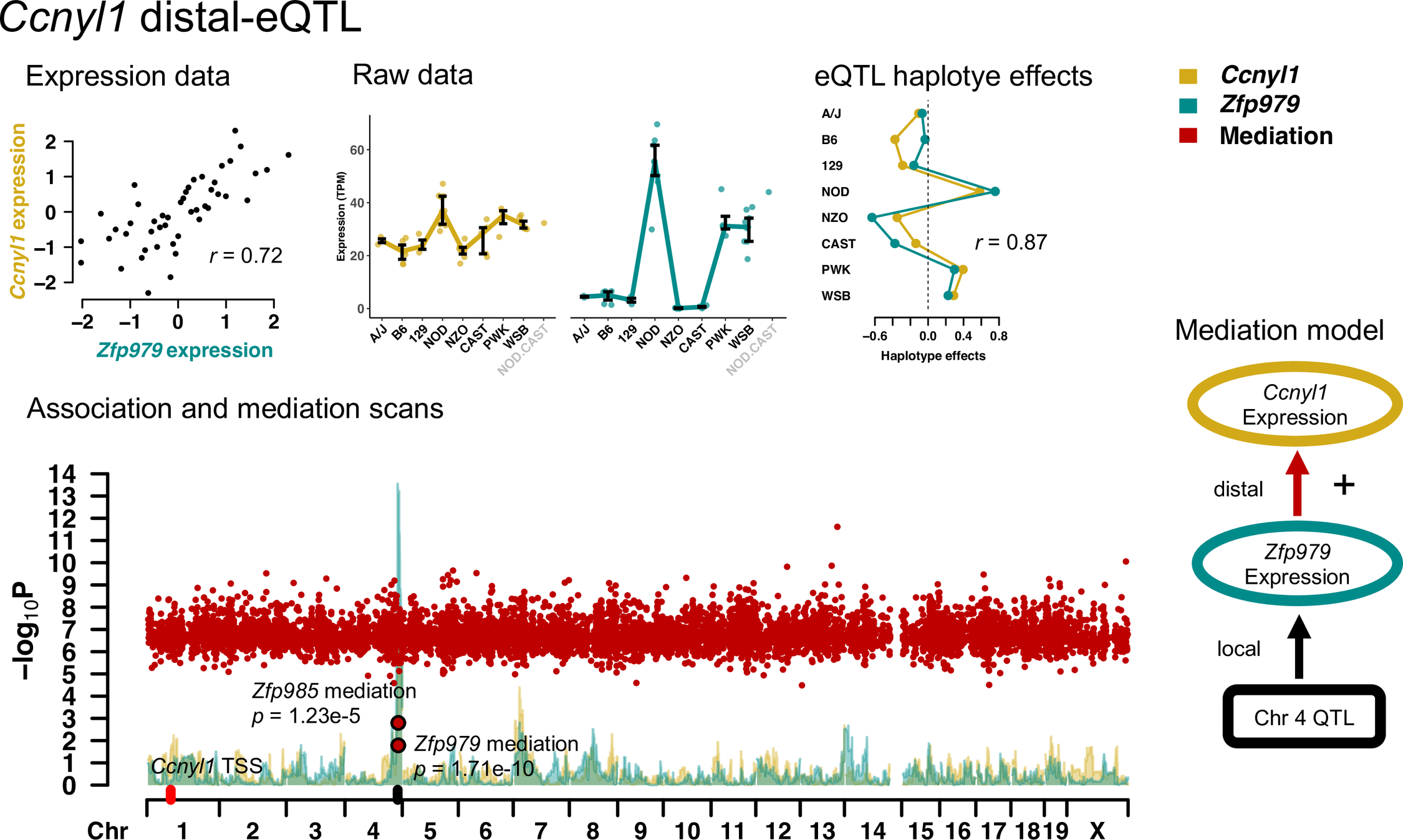

Supplement: S15 Fig — Expression of Ccnyl1 and Zfp979 are correlated (r = 0.72) in lung, which is also observed in the expression data categorized by diplotype and the haplotype effects. The distal-eQTL on chromosome 4 for Ccnyl1 corresponds closely to local-eQTL of Zfp979. Ccnyl1 is located on chromosome 1, indicated by the red tick. Zfp979 and Zfp985, both zinc finger proteins likely with DNA binding properties, are identified as strong candidate mediators of the distal-eQTL at genome-wide significance. The correlations, magnitude of effects, and mediation are consistent with the simple relationship depicted in the graph. The distal-eQTL and candidate mediators are located in a region of interest that regulates Akr1e1 expression. (TIF) [file pgen.1008537.s015.tif]

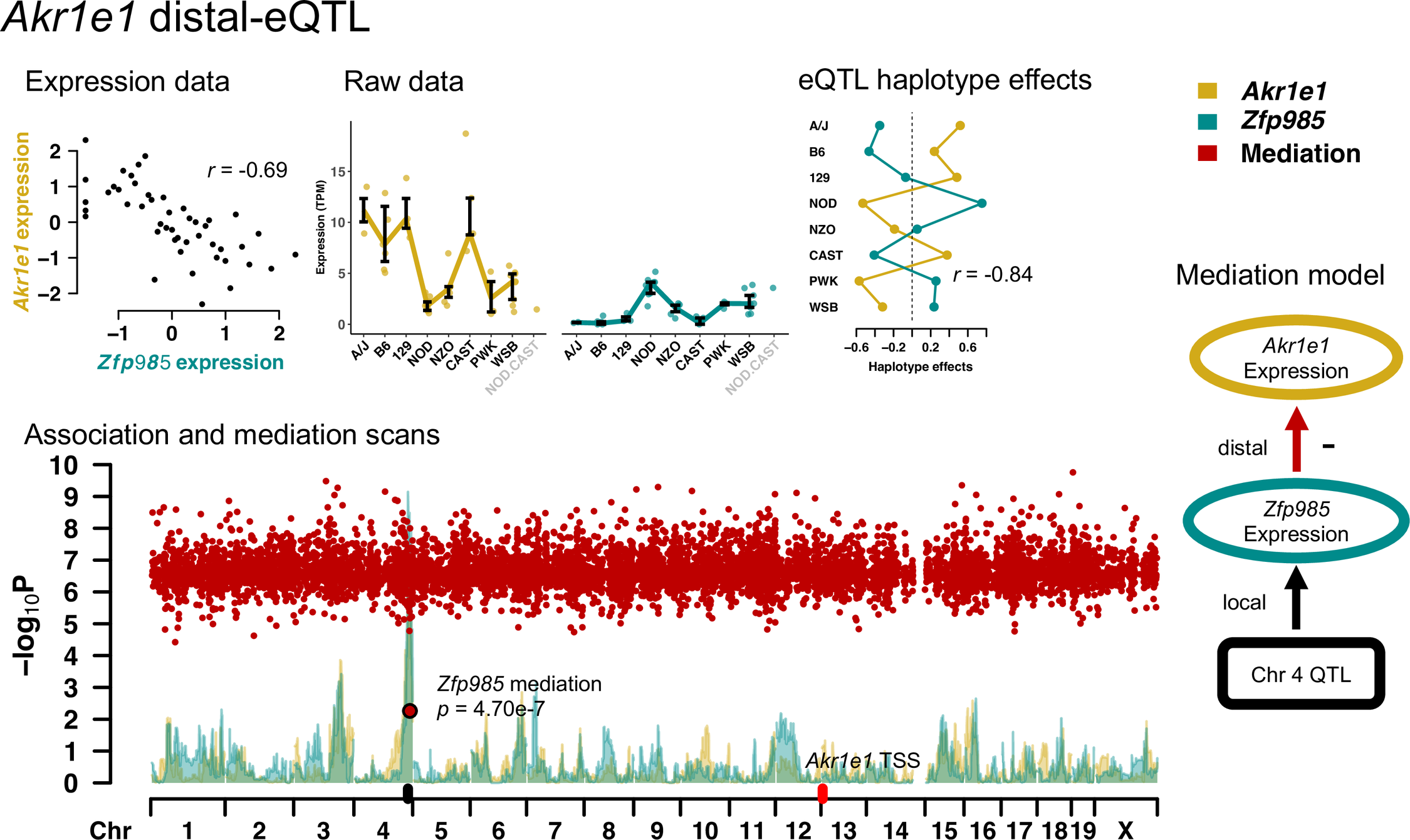

Supplement: S16 Fig — Expression of Akr1e1 and Zfp985 are anti-correlated (r = −0.69) in lung. This relationship is also observed in the expression data with bars representing the interquartile range, categorized by most likely diplotype, and the haplotype effects. The QTL and mediation scans reveal that Akr1e1, with TSS marked with a red tick on chromosome 13, possesses a distal-eQTL on chromosome 4 that is nearby the strong local-eQTL of Zfp985. The mediation scan identifies Zfp985 as a strong candidate mediator consistent with the mediation model. A more complete picture of the genetic regulation of Akr1e1 expression is pieced together by looking across all three tissues and includes a potential chromatin mediator (Fig 9). (TIF) [file pgen.1008537.s016.tif]

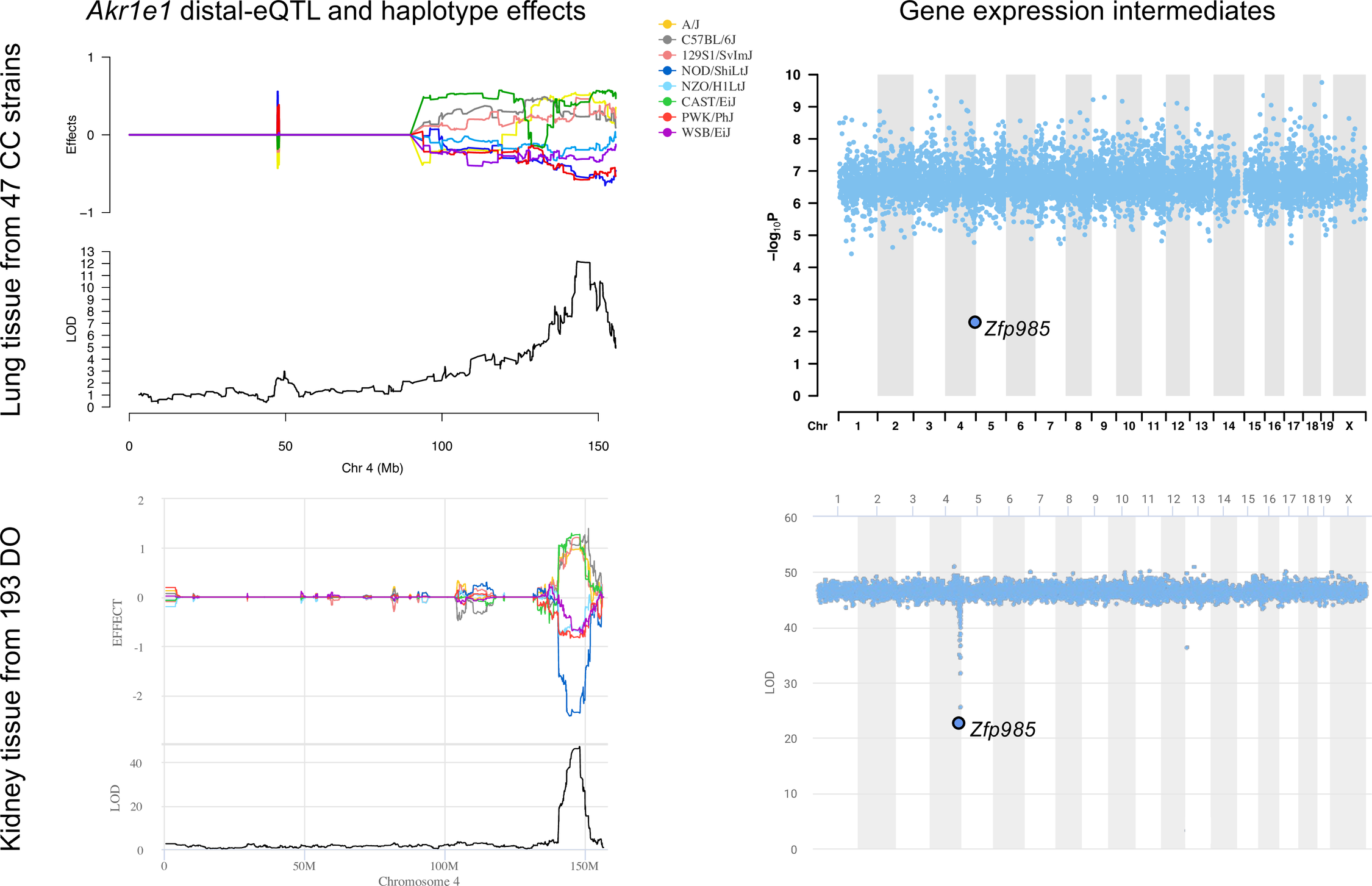

Supplement: S17 Fig — A genome-wide significant distal-eQTL was detected for Akr1e1 in liver, lung (shown here), and kidney tissues from 47 CC strains. In a larger sample of kidney tissue from outbred DO mice, the same distal-eQTL and mediation relationship were observed. As expected, the larger sample of the DO results in greater statistical significance, and confirms that the NOD effect is more strongly negative than NZO, PWK, and WSB, which the haplotype effects plots for the Zfp985 local-eQTL suggested. Notably, Zfp985 was not tested in the CC kidney because of low expression levels, though the distal-eQTL for Akr1e1 is consistent with its activity, which is here confirmed in the DO. (TIF) [file pgen.1008537.s017.tif]

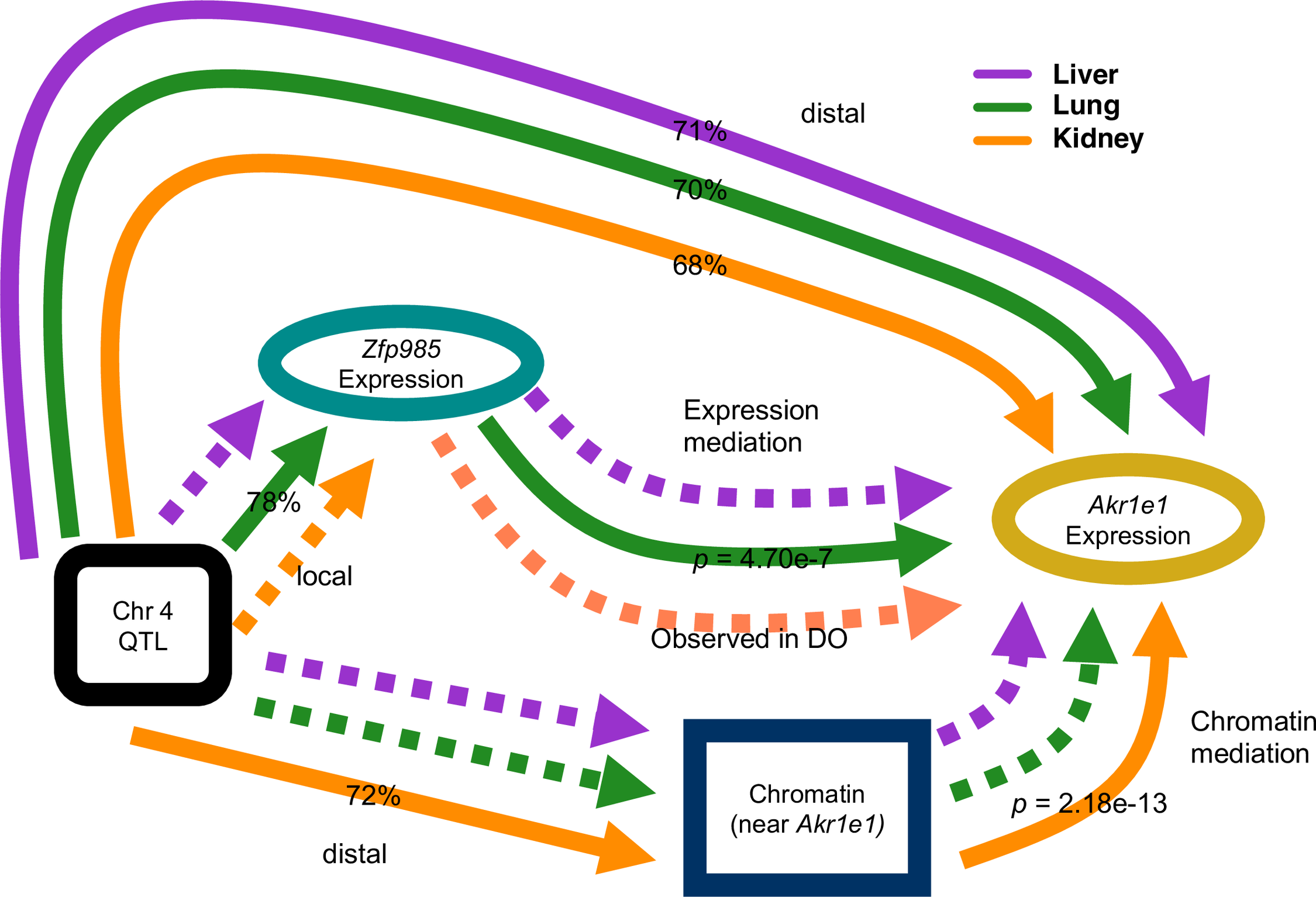

Supplement: S18 Fig — The model for the distal genetic regulation of Akr1e1 expression, described in Fig 9, was reconstructed from these observed relationships. Solid arrows were observed, whereas dashed arrows are assumed. QTL effect sizes represent the proportion of variance explained by the QTL and mediation p-values (permP) were defined using a permutation procedure. The assumed relationships are supported by the presence of the distal-eQTL in all three tissues. The Zfp985 mediator relationship in kidney, though not observed in the CC, was observed in the related DO population. (TIF) [file pgen.1008537.s018.tif]

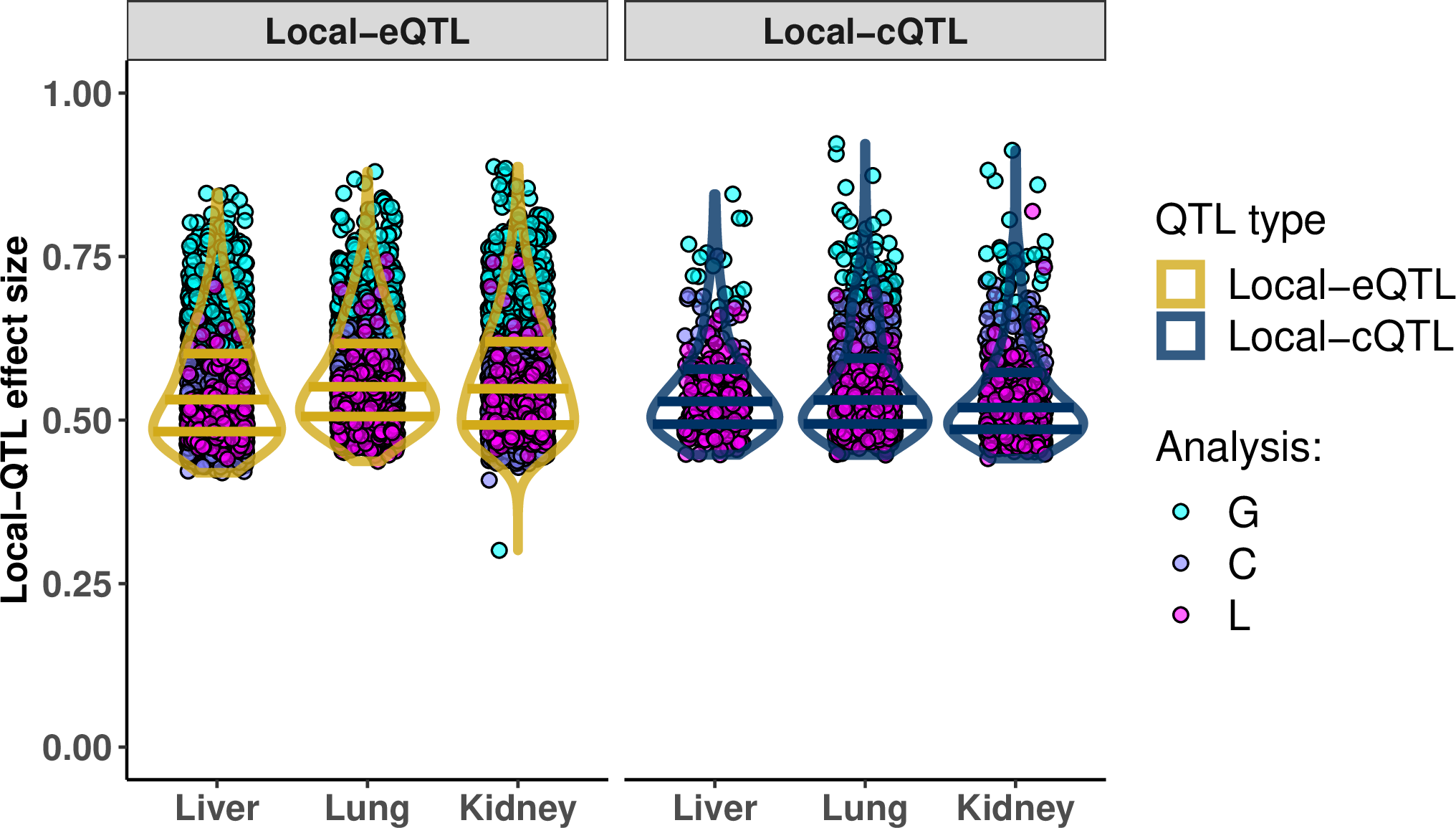

Supplement: S19 Fig — Based on ranking mapping analyses with respect to the extent of scope, local (L; magenta) to chromosome (C; plum) to genome-wide (G; cyan), the greater the scope corresponded to reduced power to detect QTL, shown in liver, lung, and kidney tissues for gene expression (yellow line) and chromatin accessibility (blue line). Each dot represents a detected local-QTL, colored according to the highest scope mapping procedure that detected it. The three horizontal bars represent the 25th, 50th, and 75th quantiles of QTL effect sizes for all local-QTL per tissue. Analysis G generally detects QTL with effect size > 60%, whereas Analyses C and L detect QTL effect sizes > 45%. Effect size estimates correspond to a fixed effects model of the QTL. (TIF) [file pgen.1008537.s019.tif]

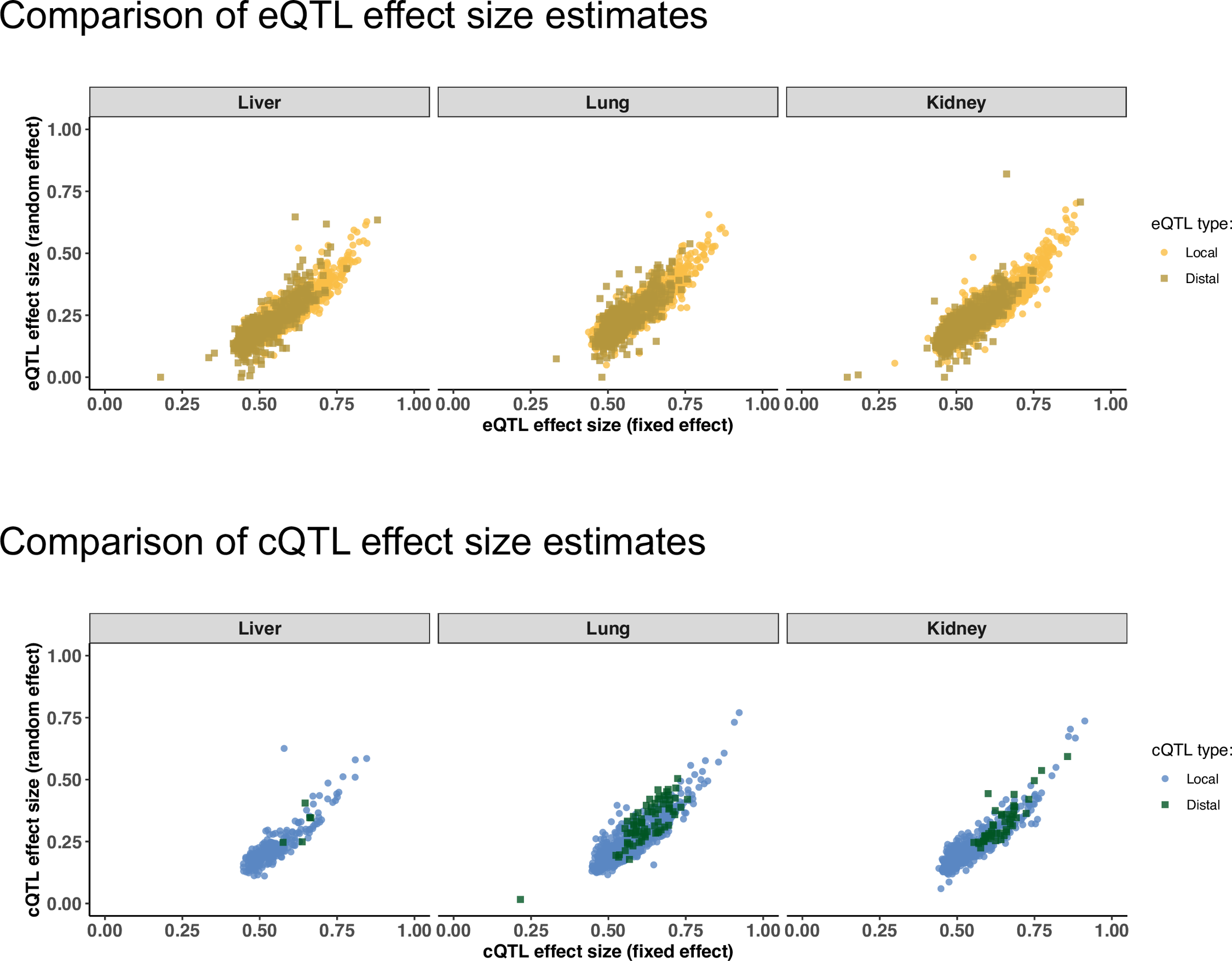

Supplement: S20 Fig — The effect size from the random effect fit is harshly penalized compared with the fixed effect estimate, likely due to a sample size of 47 mice. Notably, there are a number of distal-eQTL that are more harshly reduced by the random effects model compared with the other QTL, likely representing signals resulting from extreme observations or imbalances in founder contributions at the locus. QTL detected by Analysis G (FDR ≤ 0.1), C (FDR ≤ 0.1), and L are shown. (TIF) [file pgen.1008537.s020.tif]

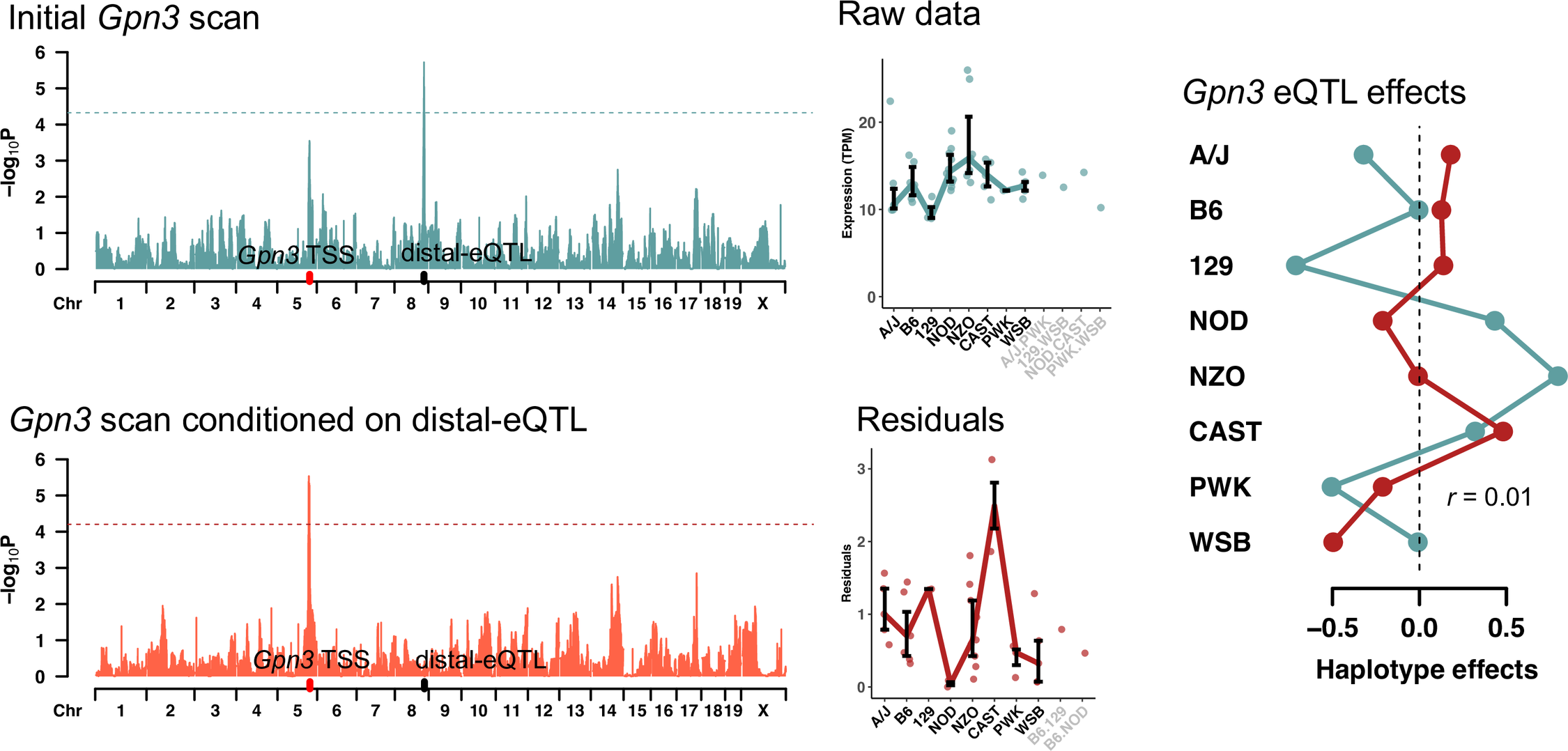

Supplement: S21 Fig — The multi-stage conditional regression approach of Analysis G allows for the detection of multiple genome-wide significant QTL, which can be appropriately incorporated into an FDR procedure across many outcomes. In this example in lung tissue, the gene Gpn3 initially has a strong distal-eQTL on chromosome 8 [top left]. Though a peak is detected near the TSS of Gpn3, it does not meet genome-wide significance. However, after conditioning on the distal-eQTL, the local-eQTL is detected [bottom left]. Horizontal dashed lines represent empirical 95% significance thresholds based on 1,000 permutations. (TIF) [file pgen.1008537.s021.tif]
