# Supplementary material for: Integrative QTL analysis of gene expression and chromatin accessibility identifies multi-tissue patterns of genetic regulation
Source: PLoS Genet. 2020 Jan 21;16(1):e1008537. doi: 10.1371/journal.pgen.1008537 (PMC7010298; doi:10.1371/journal.pgen.1008537)
Supplement: S1 Appendix — (PDF) [file pgen.1008537.s022.pdf]

S1 Appendix: CC strains

This study included a single male from the following 47 CC strains: CC001, CC002, CC003, CC004, CC005, CC006, CC007, CC010, CC011, CC012, CC013, CC015, CC016, CC017, CC019, CC020, CC021, CC023, CC024, CC025, CC027, CC028, CC029, CC030, CC031, CC032, CC033, CC035, CC036, CC037, CC038, CC039, CC040, CC041, CC042, CC043, CC044, CC045, CC046, CC049, CC053, CC055, CC057, CC059, CC061, CC062, and CC068.
