# Supplementary material for: Integrative QTL analysis of gene expression and chromatin accessibility identifies multi-tissue patterns of genetic regulation
Source: PLoS Genet. 2020 Jan 21;16(1):e1008537. doi: 10.1371/journal.pgen.1008537 (PMC7010298; doi:10.1371/journal.pgen.1008537)
Supplement: S2 Appendix — (PDF) [file pgen.1008537.s023.pdf]

# S2 Appendix: Detailed description of conditional genome-wide scans (Analysis G)

QTL analysis for a large panel of molecular traits, such as gene expression or chromatin accessibility, poses a large multiple testing problem: all loci tested for each of many traits. Analysis G employs a per-trait family-wise error rate (FWER) control and across-trait false discovery rate (FDR) control, which restricts its ability to detect multiple QTL per outcome in a non-biased manner. This bias is due to the generalized extreme value distribution (GEV), used for FWER control, being fit from the maximum statistical score per trait. To include additional strong statistical scores is potentially biasing, as would be the case when there are multiple associations above the FWER threshold, increasing the enrichment in small $p$-values, which is precisely the pattern of association FDR procedures aim to detect. To avoid this problem, a multi-stage conditional regression approach was used [1]. The procedure is described in the following steps:

1. For a given trait, conduct a genome scan according to model:

$$y_i = \mu + \text{batch}_{b[i]} + \text{QTL}_i + \varepsilon_i, \tag{1}$$

   where $y_i$ is the trait level for individual $i$, $\mu$ is the intercept, $\text{batch}_b$ is a categorical fixed effect covariate with five levels $b = 1, \ldots, 5$ representing five sequencing batches for both gene expression and chromatin accessibility and $b[i]$ denoting the batch relevant to $i$, $\varepsilon_i \sim \text{N}(0, \sigma^2)$ models the residual error, and $\text{QTL}_i$ models the genetic effect at the locus.

2. Perform permutation scans to characterize a GEV. Calculate a genome-wide error rate controlled $p$-value, $\text{permP}_\text{G}$, from the observed maximum logP of the genome scan. The $\text{permP}_\text{G}$ is stored to be used as input to an FDR procedure.

3. Specify a genome-wide $\alpha_\text{step}$ for determining whether subsequent conditional scans should be conducted for the trait. We set $\alpha_\text{step} = 0.1$. If $\text{permP}_\text{G} > \alpha_\text{step}$, no further conditional scans are conducted.

4. If $\text{permP}_\text{G} \leq \alpha_\text{step}$, steps 1-3 are repeated for an additional conditional scan of the outcome. For $j > 1$, the $j^\text{th}$ conditional scan uses the same form of alternative and null model as described in Eq 1, except with the inclusion of locus effects from the peak associations from previous stages. Generally, the alternative model for conditional scan $J$ follows as:

$$y_i = \mu + \text{QTL}_i + \sum_{j=1}^{J-1} \text{locus}_i^j + \text{batch}_{b[i]} + \varepsilon_i, \tag{2}$$

   with $\text{locus}_i^j$ representing the locus effect of the peak association from the $j^\text{th}$ stage scan of the outcome for individual $i$, and is also included in the null model of conditional scans. Now steps 2-4 are repeated.

A multi-stage conditional scan approach could have issues with over-fitting, given the data comprise 47 mice and each QTL and locus effect represents the estimation of seven fixed effects. However, permutation thresholds were found to appropriately compensate for this potential problem based on the recalculation of the GEV using permutations of the conditional scans in step 2. Fig S21 provides a clear example in which the gene *Gpn3* has a local-eQTL detected after a strong distal-eQTL is conditioned on in lung tissue.

## References

[1] Jansen R, Hottenga JJ, Nivard MG, Abdellaoui A, Laport B, de Geus EJ, et al. Conditional eQTL analysis reveals allelic heterogeneity of gene expression. Human Molecular Genetics. 2017;26(8):1444–1451. doi:10.1093/hmg/ddx043.
