# Supplementary material for: Integrative QTL analysis of gene expression and chromatin accessibility identifies multi-tissue patterns of genetic regulation
Source: PLoS Genet. 2020 Jan 21;16(1):e1008537. doi: 10.1371/journal.pgen.1008537 (PMC7010298; doi:10.1371/journal.pgen.1008537)
Supplement: S1 Table — (PDF) [file pgen.1008537.s025.pdf]

Table S1: **Number of differentially expressed genes and accessible chromatin regions detected between liver, lung, and kidney tissues at FDR** ≤ 0.1

		Tissue comparison		
		Liver/lung	Liver/kidney	Lung/kidney
Genes	Up-regulated	2,473 (20.8[a])	2,123 (17.9[a])	2,246 (18.9[a])
	Down-regulated	3,236 (27.2[a])	1,441 (12.1[a])	2,527 (21.3[a])
	Total	5,709 (48.0[a])	3,564 (30.0[a])	4,773 (40.2[a])
Chromatin regions	Increased accessibility	20,194 (19.6[b])	15,252 (12.9[b])	19,202 (17.4[b])
	Decreased accessibility	20,603 (19.7[b])	12,796 (11.4[b])	12,967 (11.2[b])
	Total	40,797 (39.3[b])	28,048 (24.3[b])	32,169 (28.6[b])

[a] Percentage of all tested genes.

[b] Percentage of all tested chromatin regions prior to merging adjacent genomic windows.
