# Supplementary material for: Integrative QTL analysis of gene expression and chromatin accessibility identifies multi-tissue patterns of genetic regulation
Source: PLoS Genet. 2020 Jan 21;16(1):e1008537. doi: 10.1371/journal.pgen.1008537 (PMC7010298; doi:10.1371/journal.pgen.1008537)
Supplement: S2 Table — (PDF) [file pgen.1008537.s026.pdf]

Table S2: **Number of genes with eQTL detected in liver, lung, and kidney tissues at FDR** ≤ 0.1

		Tissue (%)		
Procedure	eQTL type	Liver	Lung	Kidney
Analysis G	All	520 (6.2[a])	478 (4.2[a])	739 (7.3[a])
	Local[d]	400 (76.9[b])	369 (77.2[b])	601 (81.3[b])
	Distal[e]	132 (25.4[b])	112 (23.4[b])	148 (20.0[b])
Analysis C	All	2,587 (30.8[a])	2,069 (18.2[a])	3,191 (31.6[a])
	Local[d]	1,749 (67.6[c])	1,498 (72.4[c])	2,214 (69.4[c])
	Distal[e]	838 (32.4[c])	571 (27.6[c])	977 (30.6[c])
Analysis L[f]	Genome-wide FWER < 0.05	702 (8.4[a])	713 (6.3[a])	955 (9.5[a])
	Chromosome-wide FWER < 0.05	1,661 (19.8[a])	1,880 (16.6[a])	2,102 (20.8[a])

[a] Percentage of all tested genes.
[b] Percentage of genes with eQTL from Analysis G.
[c] Percentage of genes with eQTL from Analysis C.
[d] Within 10Mb upstream or downstream of gene TSS.
[e] More than 10Mb upstream or downstream of gene TSS, or on another chromosome.
[f] Not FDR controlled.
