# Supplementary material for: Integrative QTL analysis of gene expression and chromatin accessibility identifies multi-tissue patterns of genetic regulation
Source: PLoS Genet. 2020 Jan 21;16(1):e1008537. doi: 10.1371/journal.pgen.1008537 (PMC7010298; doi:10.1371/journal.pgen.1008537)
Supplement: S3 Table — (PDF) [file pgen.1008537.s027.pdf]

Table S3: **Number of genes with eQTL detected in liver, lung, and kidney tissues at FDR** ≤ 0.2

Procedure	eQTL type	Tissue (%)		
		Liver	Lung	Kidney
Analysis G	All	881 (10.5[a])	811 (7.1[a])	1,189 (11.8[a])
	Local[d]	568 (64.5[b])	522 (64.4[b])	809 (68.0[b])
	Distal[e]	339 (38.5[b])	301 (37.1[b])	411 (34.6[b])
Analysis C	All	4,699 (55.9[a])	3,675 (32.4[a])	5,469 (54.2[a])
	Local[d]	2,519 (53.6[c])	2,213 (60.2[c])	3,099 (56.7[c])
	Distal[e]	2,180 (46.4[c])	1,462 (39.8[c])	2,370 (43.3[c])

[a] Percentage of all tested genes.
[b] Percentage of genes with eQTL from Analysis G.
[c] Percentage of genes with eQTL from Analysis C.
[d] Within 10Mb upstream or downstream of gene TSS.
[e] More than 10Mb upstream or downstream of gene TSS, or on another chromosome.
