# Supplementary material for: Integrative QTL analysis of gene expression and chromatin accessibility identifies multi-tissue patterns of genetic regulation
Source: PLoS Genet. 2020 Jan 21;16(1):e1008537. doi: 10.1371/journal.pgen.1008537 (PMC7010298; doi:10.1371/journal.pgen.1008537)
Supplement: S4 Table — (PDF) [file pgen.1008537.s028.pdf]

Table S4: **Number of chromatin accessibility sites with cQTL detected in liver, lung, and kidney tissues at FDR** ≤ 0.1

		Tissue (%)		
Procedure	cQTL type	Liver	Lung	Kidney
Analysis G	All	17 (0.1[a])	114 (0.5[a])	59 (0.3[a])
	Local[d]	16 (94.1[b])	78 (68.4[b])	39 (66.1[b])
	Distal[e]	1 (5.9[b])	36 (31.6[b])	20 (33.9[b])
Analysis C	All	39 (0.3[a])	226 (0.9[a])	130 (0.7[a])
	Local[d]	35 (89.7[c])	186 (82.3[c])	98 (75.4[c])
	Distal[e]	4 (10.3[c])	40 (17.7[c])	32 (24.6[c])
Analysis L[f]	Genome-wide FWER $<$ 0.05	70 (0.6[a])	244 (1.0[a])	149 (0.8[a])
	Chromosome-wide FWER $<$ 0.05	299 (2.6[a])	876 (3.6[a])	616 (3.4[a])

[a] Percentage of all tested chromatin regions.

[b] Percentage of genes with cQTL from Analysis G.

[c] Percentage of genes with cQTL from Analysis C.

[d] Within 10Mb upstream or downstream of chromatin region midpoint.

[e] More than 10Mb upstream or downstream of chromatin region midpoint, or on another chromosome.

[f] Not FDR controlled.
