# Supplementary material for: Integrative QTL analysis of gene expression and chromatin accessibility identifies multi-tissue patterns of genetic regulation
Source: PLoS Genet. 2020 Jan 21;16(1):e1008537. doi: 10.1371/journal.pgen.1008537 (PMC7010298; doi:10.1371/journal.pgen.1008537)
Supplement: S5 Table — (PDF) [file pgen.1008537.s029.pdf]

Table S5: **Number of chromatin accessibility sites with cQTL detected in liver, lung, and kidney tissues at FDR** ≤ 0.2

		Tissue (%)		
Procedure	cQTL type	Liver	Lung	Kidney
Analysis G	All	20 (0.2[a])	220 (0.9[a])	113 (0.6[a])
	Local[d]	18 (90.0[b])	116 (52.7[b])	55 (48.7[b])
	Distal[e]	2 (10.0[b])	105 (47.7[b])	58 (51.3[b])
Analysis C	All	62 (0.5[a])	309 (1.3[a])	249 (1.4[a])
	Local[d]	50 (80.6[c])	238 (77.0[c])	149 (59.8[c])
	Distal[e]	12 (19.4[c])	71 (23.0[c])	100 (40.2[c])

[a] Percentage of all tested chromatin regions.
[b] Percentage of genes with cQTL from Analysis G.
[c] Percentage of genes with cQTL from Analysis C.
[d] Within 10Mb upstream or downstream of chromatin region midpoint.
[e] More than 10Mb upstream or downstream of chromatin region midpoint, or on another chromosome.
