# Supplementary material for: Integrative QTL analysis of gene expression and chromatin accessibility identifies multi-tissue patterns of genetic regulation
Source: PLoS Genet. 2020 Jan 21;16(1):e1008537. doi: 10.1371/journal.pgen.1008537 (PMC7010298; doi:10.1371/journal.pgen.1008537)
Supplement: S6 Table — (PDF) [file pgen.1008537.s030.pdf]

Table S6: **Number of genes with chromatin mediation of local-eQTL in liver, lung, and kidney tissues**

		Tissue (%)		
Procedure	Significance	Liver	Lung	Kidney
Analysis L[b]	genome-wide	13 (0.8[a])	42 (2.2[a])	21 (1.0[a])
	chromosome-wide	35 (2.1[a])	106 (5.6[a])	66 (3.1[a])

[a] Percentage of genes with a chromatin mediator that meet chromosome-wide significance for a local-eQTL through Analysis L.

[b] 10Mb upstream or downstream of the gene TSS with local-eQTL that meets chromosome-wide significance through Analysis L. Additionally, chromatin mediator must possess a local-cQTL that meets chromosome-wide significance through Analysis L.
