# Supplementary material for: Integrative QTL analysis of gene expression and chromatin accessibility identifies multi-tissue patterns of genetic regulation
Source: PLoS Genet. 2020 Jan 21;16(1):e1008537. doi: 10.1371/journal.pgen.1008537 (PMC7010298; doi:10.1371/journal.pgen.1008537)
Supplement: S7 Table — (PDF) [file pgen.1008537.s031.pdf]

Table S7: **Genes with distal-eQTL with gene mediators detected in lung and kidney tissues**

Tissue	Gene	Chr	Mediator gene	Chr	$permP_G^m$
Lung	*Akr1e1*	13	*Zfp985*	4	4.70e-07
	Ccnyl1	1	*Zfp979*	4	1.71e-10
			Zfp985	4	1.23e-05
	Man2c1	9	*Vash1*	12	1.77e-06
	Rbm46	3	*Gatm*	2	1.06e-02
Kidney	*C330018D20Rik*	13	*Depdc1b*	18	9.97e-04
	Fbln5	12	*Crym*	7	3.67e-02
	Gcdh	8	*Dmgdh*	13	8.96e-04
	Oscp1	4	*Slc25a34*	4	3.27e-03
