# Supplementary material for: Integrative QTL analysis of gene expression and chromatin accessibility identifies multi-tissue patterns of genetic regulation
Source: PLoS Genet. 2020 Jan 21;16(1):e1008537. doi: 10.1371/journal.pgen.1008537 (PMC7010298; doi:10.1371/journal.pgen.1008537)
Supplement: Attachment — Submitted filename: plosG_reviews.docx [file pgen.1008537.s046.docx]

Reviews

These reviews pertain to the article PGENETICS-D-19-00860: “*Integrated QTL analysis of gene expression and chromatin accessibility identify multi-tissue patterns of genetic regulation*”. The authors use 47 mouse strains of the Collaborative Cross to integrate QTL effects for the genetic regulation of gene expression and chromatin accessibility in liver, kidney and lung tissues. They report on both tissue-specific QTL and inter-tissue QTL identified via similarity analysis of haplotype effects, report novel correlated haplotype effects for local and distal QTL across tissues, and demonstrate specific causal candidate genes of QTL.

This study, which is well written, presents two major merits. Multi-parental populations (MPPs) have become resources of interest in quantitative genetics and genomics in which whole genome sequencing of large collections of recombinant inbred lines is not readily feasible. This study presents the possibility to examine co-localizing QTL effects using founder haplotypes. I think that the method as is, and with potential future refinements, makes integrative QTL methods stronger, particularly in dealing with multiple founder haplotypes. Secondly, the study joins a few other studies that are coopting mediation analysis (a concept with roots in psychology) to genomics. In particular, a statistically rigorous approach used here that takes into account size of QTL effects to identify significant relationships between eQTL and cQTL (i.e. ‘anyQTL’), potentially has wide application in the search for causal genetic variants of important traits, including disease traits. I recommend publication of this report with minor revisions.

1. Line 40. Expression patterns can be sex-specific in many species. It’s useful to add the rationale for using only males in this study. Because the aim of the study is to characterize the relationship between gene expression and chromatin state dynamics, one would probably expect a different outcome if females were used.
2. Lines 62 – 64. The discordance between number of DEGs (3,564 – 5,709) and DARs (28,048 – 40,797) is striking. Is there a reason for this difference? The authors may briefly comment either here or in the discussion.
3. Line 79. The definition of a local QTL as one that occurs within 10 Mb of TSS needs justifying with a citation or data or reasoning. In mouse, would 10 Mb be equivalent to say, 10 cM in genetic distance, which appear as relatively short for the definition of “local”?
4. Lines 83 and 96-97. Why is the number of genes included in eQTL much lower for liver tissue compared to other organs (i.e. 8,401 vs >10,000 for eQTL, and 11448 vs >17,900 for DARs)? If more of liver genes and DARs for liver were filtered out, why? Does this relatively low starting number of included liver genes affect downstream comparisons such as tissue pairwise comparisons in Tables S1 and S2 and Fig. 8, for example?
5. Line 98-99. Why is there substantially fewer cQTL relative to eQTL in general? And, given vast numbers of DARs relative to DEGs?
6. Lines 114 – 115. This generally looks true. However, the plot for liver (Fig. S8B) would suggest the numbers much lower in liver which would relate to stated lower gene count in liver analyses to begin with.
7. Lines 138 – 139. What do the authors attribute the lack of negatively correlated eQTL pairs? Could there be pervasive transgressive QTL or QTL acting in opposite directions that cancel each other out leading to non-detection? Is there a sex-ratio distortion segregating in the CC which could contribute to this finding?
8. Line 147. “…levels..” “level”?
9. Lines 441 – 442. There is a huge difference in literature on filtering thresholds for expression data, making it really hard for newer folks to decide thresholds on their data. This is a good place to give rationale for selecting these levels.
10. Line 459. Same as in point 9 above.

General comments

1. Introduction section. Since mediation analysis features so prominently in the manuscript, and a few citations are provided (due to the nascent stage of this application in genomics), the authors may add a brief introduction and significance of this concept in MPPs.
2. Judging by the frequency with which I have had to look at supplementary materials raises the question of whether a good proportion of the important messages are hidden there? Within the constraints of journal requirements some figures could be promoted into the main text – may be one panel to group a few messages, particularly in the QTL section.
3. References. Some references are in sentence case (e.g. Ref. 34, others in a different format (e.g. Refs. 22, 29). Check the whole section.
4. Refences. Ref. 31 has repeated author list.
5. Figure 1. May add text to plates showing founder haplotypes (first object top-left). Otherwise, the message is not clear at a glance, neither in the caption.
6. Figure 8. The discussion may benefit from a short explanation why liver has too few cQTL compared to the other tissues. Also, can we say that the liver and kidney show little mediation through expression compared to lung? Why would this be the case?
