# Supplementary material for: Integrative QTL analysis of gene expression and chromatin accessibility identifies multi-tissue patterns of genetic regulation
Source: PLoS Genet. 2020 Jan 21;16(1):e1008537. doi: 10.1371/journal.pgen.1008537 (PMC7010298; doi:10.1371/journal.pgen.1008537)
Supplement: Attachment — Submitted filename: response_to_editors.docx [file pgen.1008537.s047.docx]

**Keele & Quach et al. (2019+) “Integrative QTL analysis of gene expression and chromatin accessibility identifies multi-tissue patterns of genetic regulation” PGENETICS-D-19-00860**

We would like to thank the editors for their efforts in coordinating this review and the reviewers for their insightful comments on our original manuscript. We have addressed each of these concerns, as detailed below. We have re-submitted a revised manuscript reflecting the indicated updates. We believe this has greatly improved the paper, and we hope the editors agree that it is now suitable for publication.

**Reviewer 1**

**Minor revisions:**

1. Line 40: **“Here we use a sample composed of a single male mouse from 47 CC strains...”** Expression patterns can be sex-specific in many species. It’s useful to add the rationale for using only males in this study. Because the aim of the study is to characterize the relationship between gene expression and chromatin state dynamics, one would probably expect a different outcome if females were used.

We appreciate this comment from Reviewer 1 and agree that there is strong evidence that gene expression (*eg*. Chick *et al.* 2016), and likely chromatin state, vary between the sexes. As such, these results are male-specific, which we now further emphasize at the beginning of the Results. It was decided to experimentally control for sex effects by using only males to avoid an experiment of 188 animals and to constrain costs as well as the at-times limited availability of CC mice. Using only males therefore limits our ability to generalize across sexes but does at least remove sex as a confounding factor.

2. Lines 62 – 64: **“Differential expression (DE) and differentially accessible region (DAR) analysis were performed between the three tissues (S1 Table), and revealed between 3,564 - 5,709 DE genes and 28,048 - 40,797 DARs (FWER < 0.1).”** The discordance between number of DEGs (3,564 – 5,709) and DARs (28,048 – 40,797) is striking. Is there a reason for this difference? The authors may briefly comment either here or in the discussion.

In general, there is no expectation as to the relationship between the number of DE genes and DARs, as gene expression can be altered without a change in chromatin accessibility, and chromatin accessibility can be altered without an immediate change in gene expression. In the case here, we would expect a lot of differences at the chromatin level between tissues, as the gene regulatory program defined by accessible chromatin reflects very distinct differentiation paths for cells in each tissue. Multiple elements can regulate single genes, especially genes that are cell- and tissue-specific, often following finely controlled expression patterns. Accessible chromatin also identifies poised regulatory elements that may not be active in current conditions, but help the cell up or down regulate certain genes in response to changing cellular conditions or external stimuli. Thus, the large number of DARs reflects both the large number of identified DE genes as well as highly divergent functions of constituent cells that are poised to adopt future expression patterns quickly. We do emphasize that DE genes and the DARs, constrained to the promoters of the genes, are largely concordant with each other in comparison to discordant (see Fig S2).

3. Line 79: **“…at the level of the local region of a gene, defined as within 10Mb of the gene transcription start site (TSS)...”** The definition of a local QTL as one that occurs within 10 Mb of TSS needs justifying with a citation or data or reasoning. In mouse, would 10 Mb be equivalent to say, 10 cM in genetic distance, which appear as relatively short for the definition of “local”?

In mouse, 10Mb is approximately 5cM, since on average 2Mb ≈ 1cM (http://www.informatics.jax.org/mgihome/other/mouse_facts1.shtml). However, although 10Mb/5cM would be short in a traditional mouse cross, such as an F2 intercross or backcross where few recombinations have occurred and LD is very strong and mapping resolution is coarse, it is far more reasonable in the CC, where a greater number of generations have broken up the LD blocks and resolution is much higher. Indeed, in this context we view +/- 10Mb as being a fairly lenient local window, though notably, it has been used in the analysis of the DO (Chick *et al.* 2016), which possess more recombination events (finer LD structure) and generally have larger sample sizes, all of which should improve mapping resolution in the DO compared to CC. Local QTL may represent *cis* or non-*cis* regulation, but based on Fig 2B and Fig S6, the 10Mb local window appears to capture the majority of strong QTL in the neighborhood of the gene TSS and chromatin window midpoint. We have added additional text to the Methods to this effect, justifying our definition.

4. Lines 83 and 96-97: **“…8401 for liver, 11357 for lung, and 10092 for kidney…”** and **“…11448 in liver, 96 24426 in lung, and 17918 in kidney…”**. Why is the number of genes included in eQTL much lower for liver tissue compared to other organs (i.e. 8,401 vs >10,000 for eQTL, and 11448 vs >17,900 for DARs)? If more of liver genes and DARs for liver were filtered out, why? Does this relatively low starting number of included liver genes affect downstream comparisons such as tissue pairwise comparisons in Tables S1 and S2 and Fig. 8, for example?

The divergent number of genes and accessible regions used for QTL analyses across tissues is likely due to both technical and biological reasons. As stated in the Methods, we filter out lowly expressed genes and accessible regions with low signal across the majority of the samples. This is done to increase our power to detect QTL by reducing the number of tests and eliminating genes/regions for which QTL cannot be reliably detected. The variable numbers of genes and regions from each tissue that are filtered will affect the total number of QTL that can be potentially found. The differences may be due to distinct biology in those tissues that requires more or less genes to be expressed to perform necessary functions, and requiring more or less regulatory elements to direct the expression program. This variability may also be due to technical reasons, with certain assays able to more accurately detect low signals in some tissues compared to others, though we do not see systematic evidence of that in our data. We do note that in the GTEx project (<https://gtexportal.org/home/>), different tissues have varying number of detected genes with *cis*-eQTL (eGenes) despite the same number of samples. For example, liver (n=208 samples) has 5,734 eGenes, while cerebellum (n=209) has 11,240 eGenes. Thus, genetic effects on expression and chromatin accessibility may vary widely across different tissues.

5. Line 98-99: **“Overall, there were substantially fewer cQTL detected compared to eQTL for all tissues (Fig 2B; S4 Table, S5 Table).”** Why is there substantially fewer cQTL relative to eQTL in general? And, given vast numbers of DARs relative to DEGs?

We agree that it may not be obvious to the reader why there is this imbalance in QTL detection, and we have added text to the Discussion related to this point (Discussion subsection “Fewer detected cQTL than eQTL”).

6. Lines 114 – 115: **“Consistent with previous studies (e.g., [38]), the CAST and PWK haplotypes had higher magnitude effects compared to the classical inbred strains. This pattern was observed for both local- and distal-eQTL (S8 FigA) and local-cQTL; the numbers of detected distal-cQTL were too low to produce clear trends (S8 FigB).”** This generally looks true. However, the plot for liver (Fig. S8B) would suggest the numbers much lower in liver which would relate to stated lower gene count in liver analyses to begin with.

As Reviewer 1 has noted, liver had the fewest genes and chromatin windows analyzed after quality control filtering. Please see the related responses above to comments 4 and 5. For Fig S8, our intent was only to remark on the trends in the effects, where it is known that alleles from CAST and PWK, representing more genetically distinct founders, often have more extreme effects in the CC and DO. These trends are clear in the eQTL and local-cQTL, but the number of distal cQTL are too few to show stable trends, most notably for liver, but also lung and kidney as well. The inability to see such trends is very likely due to the lower numbers of distal-cQTL, seen most obviously in liver, likely reflecting the reduced number of chromatin regions analyzed.

7. Lines 138 – 139: **“No significantly negatively correlated QTL pairs were detected, after accounting for multiple testing (FDR < 0.1).”** What do the authors attribute the lack of negatively correlated eQTL pairs? Could there be pervasive transgressive QTL or QTL acting in opposite directions that cancel each other out leading to non-detection? Is there a sex-ratio distortion segregating in the CC which could contribute to this finding?

Reviewer 1 raises an interesting question about the potential for linked transgressive eQTL or cQTL within a tissue. For example, there may exist a B6 variant that drives an increase in a molecular trait (*eg.* gene expression, chromatin accessibility) and another nearby linked B6 variant that drives a decrease in the same trait. In most cases, the variants would be in LD and would cancel out each other’s effect, and there would likely be few individuals with the recombination event that decouples the two QTL. We agree that type of transgression is possible, but we are not able to determine relationships like this within a single tissue.

Instead in this work, we pair QTL *across* tissues and determine whether founder haplotype effects are correlated. We expect that these would be positively correlated in most cases, which is consistent with the same causal genetic variants being active in both tissues. In that context, anti-correlated QTL would represent something like the QTL in B6 increasing the molecular trait in liver, but decreasing the trait in lung. This may reflect tissue-specific QTL with transgressive effects, but only when viewed across tissues, similar to the paired QTL for *Slc44a3* that in liver that has a different effect compared to lung and kidney (see Fig 3B). The fact that we see an excess of positively correlated QTL pairs suggest that, overall, the genetic regulation that we are powered to detect has largely consistent effects across these tissues.

Determining the specific causal variants underlying QTL is not obvious, particularly given the LD structure of the CC population. However, using parsimony, positively correlated QTL could represent the same functional variants active in multiple tissues, whereas uncorrelated (*Pik3c2g*) and even transgressive (*Slc44a3*) QTL could represent genetic variants whose effects substantially vary in different tissues. We have added a partial sentence at the end of the “Multi-tissue QTL pairs share similar haplotype effects across tissues” section of the Results to further emphasize our interpretations. Finally, these data represent only males and thus cannot shed light on potential sex-ratio distortions in the CC.

8. Line 147. “…levels..” “level”?

Correction made.

9. Lines 441 – 442: **“TMM-normalized TPM values ≤ 1 and chromatin windows with normalized counts ≤ 5 for ≥ 50% of samples were excluded.”** There is a huge difference in literature on filtering thresholds for expression data, making it really hard for newer folks to decide thresholds on their data. This is a good place to give rationale for selecting these levels.

We agree with Reviewer 1 that processing and filtering these high-throughput phenotypes varies greatly across the literature, which can make it challenging to know what steps are best or necessary for a given experiment or data type. Some of this variability likely reflects the context and limits of specific experiments and data. Towards making this process more transparent and clearer to readers, we have added a small statement clarifying that we filter lowly expressed genes in order to avoid QTL that result from highly influential non-zero values in the presence of a significant portion of near-zero values. We have also cited (Chick *et al.* 2016), which used a similar 50% low expression cutoff.

10. Line 459: **“Windows in which > 70 % of samples had a CPM ≤ 1 were removed.”** Same as in point 9 above.

Following similar reasoning to our response for the previous comment from Reviewer 1, we further clarify the rationale behind the filtering used for the differential tissue analysis. Genes and chromatin windows with low or no counts across sample libraries provide little evidence for differential gene expression (DGE) and differentially accessible chromatin regions (DAR) across tissues. Including these outcomes in the differential tissue analysis would increase the multiple testing burden, ultimately reducing the power to detect DGE and DAR. Given that each tissue type is measured for the same samples, having reads for a gene or window detected for at least 70% of samples across tissues approximately requires samples from at least two of the three tissues to have non-zero measurements before being considered for differential tissue analysis.

**General comments:**

1. Introduction section. Since mediation analysis features so prominently in the manuscript, and a few citations are provided (due to the nascent stage of this application in genomics), the authors may add a brief introduction and significance of this concept in MPPs.

We have expanded the 4^th^ paragraph of the Introduction to provide a little more thought to the potential strengths of MPPs for mediation analysis specifically, though as Reviewer 1 notes, its use in genomics generally, including MPPs, is fairly new.

2. Judging by the frequency with which I have had to look at supplementary materials raises the question of whether a good proportion of the important messages are hidden there? Within the constraints of journal requirements some figures could be promoted into the main text – may be one panel to group a few messages, particularly in the QTL section.

We appreciate this suggestion from Reviewer 1 and have expanded Fig 2, which previously included only the mapping results from all tissues to include comparisons between lung and kidney in added panels. Within the text, we now point to this figure, as well as the Supplement for the complete set of figures for all the tissues. We feel this has made Fig 2 more informative, as well as reduced the need to frequently refer to Supplemental figures.

3. References. Some references are in sentence case (e.g. Ref. 34, others in a different format (e.g. Refs. 22, 29). Check the whole section.

We have corrected the references and double checked that they all comply with the citation format specified by PLoS journals.

4. Refences. Ref. 31 has repeated author list.

Correction made.

5. Figure 1. May add text to plates showing founder haplotypes (first object top-left). Otherwise, the message is not clear at a glance, neither in the caption.

We have now added a legend to Fig 1 that matches the founder strains to their letter code in the funnel graphic.

6. Figure 8. The discussion may benefit from a short explanation why liver has too few cQTL compared to the other tissues. Also, can we say that the liver and kidney show little mediation through expression compared to lung? Why would this be the case?

As discussed above in responses to comments 2 and 4, detection of QTL is dependent on not only sample size, but also biological and technical factors that are difficult to quantify with these data. We would be hesitant to make strong biological conclusions on differences in numbers of QTL or mediators, or the lack thereof, in any given tissue relative to the other tissues, based on the data in this study. While it is possible that liver and kidney do have reduced mediation compared to lung, we cannot address this question. If there is a true biological difference in mediator usage between tissues, it likely reflects tissue-specific multi-level regulatory programs, a complex subject that is outside the scope of the current study. We agree that this is an interesting point, and we have added a section in the Discussion related to this.

**Reviewer 2**

**Major comments:**

1. Page 18, Mediation analysis, **“the eQTL is always included in the alternative model but not the null model.”** I am not sure why not instead exclude the mediator in the null model. It seems that excluding the eQTL in the null model is to test partial mediation vs. full mediation. Based on my understanding, mediation analysis is to test the red mediation path in Fig 6 [Refs 9, 56], not the yellow eQTL path. Then in S3 Appendix, **“Instead of permuting the trait outcome, the pairing of trait to QTL is maintained, and the mediator is permuted.”** It seems the mediator was tested. It is confusing, and clarification should be given.

We agree that this was unclear and have rewritten the mediation analysis section of the methods. The revised section starts by providing a clearer description of steps used in traditional mediation, including the conditions for partial vs full mediation, and then outlines our approximation to that approach, based on more clearly cited literature, written in a way that allows more direct comparison with the traditional scheme. The reviewer is correct that our genome-wide mediation is more similar to testing for full mediation, and we hope this is now more explicit. Additionally, we have re-worded the later technical details of this section, namely the specification of null and alternative models, and have updated the relevant section in the supplement.

2. The permutation-based p-values were calculated using a nonstandard approach based on generalized extreme value distribution (GEV). Is this method designed for genetically correlated samples? Is it more efficient than simply comparing the observed statistic with the null statistics? Is there a way to empirically prove that this approach works for the analyzed data?
   In S3 Appendix, **“An FDR adjustment is not used with mediation because it is only evaluated for detected QTL, which violates the uniform null distribution expectations of FDR.”** At least comparing the observed statistic with the null statistics can control FDR, which is more liberal than family-wise error rate (FWER) control. It can be more powerful, especially when the sample size is only 47, and the testing is genome-wide.

The GEV is to maxima (or minima) what the Gaussian distribution is to sums (or means): it is the distribution to which those quantities asymptotically converge. In the context of a genome-wide permutation scan, the statistic recorded for each permutation is a maximum (or in the mediation scans, a minimum) of a large number of random variables (the per-locus or per-mediator tests). The distribution of those maxima is most often used directly, with data quantiles used to approximate theoretical threshold quantiles (*eg*. the upper 0.05 quantile). However, since this distribution will typically take on the form of a GEV, it is orders of magnitude more efficient to fit a GEV distribution (which has three parameters) and then use the resulting fitted distribution to estimate the threshold quantiles via its cumulative distribution function. This particular use of the GEV was first described in (Valdar *et al.* 2006) (see Figure 2 of that paper and the related section of its Methods, titled: “Assessing statistical significance using generalized extreme value distributions”), but a closely related use of the GEV was also described earlier in (Dudbridge and Koeleman 2004)

For mediation scans we use the GEV to control the FWER, similar to as done with the QTL scans. As Reviewer 2 suggests, we could use the null statistics from the permutations of either the QTL or mediation scan to determine a significance threshold that controls the FDR, which is more lenient than controlling FWER, for that specific scan. This is an interesting suggestion; however, it may be best implemented as part of an additional rigorous study on the power and error properties of mediation analysis rather than added to this manuscript. For this study, we apply an FWER control per each outcome (for QTL) and each eQTL (for mediation), as for each, we are performing many tests in a scan and need to tightly control false positives. Our assumption is that there are not many QTL for an outcome and not many mediators for an eQTL, particularly given the power constraints of the data.

The FDR approach in our view is most useful when it is expected that there are many statistically significant signals, as we do when we employ FDR control across all the outcomes in the QTL scans. Given the additional complications already present in genome-wide mediation analysis and the potential for false positives, we are hesitant to use a more lenient correction. In the S3 Appendix, we added the following sentences: “Notably, we use the genome-wide and chromosome-wide measurement of molecular traits, the vast majority of which are ineligible to be mediators of the eQTL due to location, to characterize empirical error distributions for the mediator test, and thus derive FWER-controlling thresholds. This approach is statistically stringent, given that mediators must be local to the eQTL, which is appealing in terms of controlling false positives that could result in biologically-complex data.”

3. The R package miQTL seems to lack helpfile/tutorial for functions related to mediation analysis. Those would make the scheme of mediation analysis clearer. Or in the paper, providing a concise algorithm to list each step of the mediation analysis would help. Separating gene-gene and chromatin-gene mediation analyses would help further. Right now, the methods of mediation analysis are not described clearly.

Reviewer 2 is correct in noting that the miQTL R package lacked documentation for the functions related to mediation analysis at the time this manuscript was reviewed. We have now added documentation for those functions and have also included supplemental R code files (see Files S26-31) that generate the figures and results included in the text from the pre-processed data, which we have provided at figshare (doi:10.6084/m9.figshare.9985514), and act as a small tutorial for the miQTL package.

Please see response to Reviewer 2’s Major Comment 2. We have also included more details from the S3 Appendix to specifically describe the required steps for mediation, as well as attempted to provide insights and reasoning behind those steps.

**Minor comments:**

1. Fig 1, “ATAC-seq” was misspelled as “ATATC-seq.”

The correction has been made.

2. Line 17, Ref 9 seems mis-cited. It is not related to “ribosome occupancy with protein abundances.”

Citation moved to the next sentence (was next to the wrong Battle2015 paper).

3. Fig S1, caption, “liver (pink).” The pink color seems to be purple from non-color-blind eyes.

Liver is now described as purple.

4. The captions for the supplementary figures shown in the main text file and the supplementary file do not match.

The discrepancies have been corrected.

**Reviewer 3**

**Comments on mediation analysis:**

1. The authors use mediation analysis to identify eQTL and chromatin-QTL signals that may result from the same causal variant. The authors suggest that a significant result in the mediation analysis implies that the chromatin peak regulates the gene. In fact, there are other explanations for this finding (reverse causality, pleiotropy, multiple linked causal variants). The authors refer to such issues in the Methods (Page 18, line 656). This caveat should be discussed explicitly in the main text.

We have added further detail on the caveats and limitations of the mediation analysis to the relevant paragraph in the Discussion. We emphasize that though we use the QTL effect size to identify relationships that are consistent with the proposed model, there is no way to actually experimentally control the relationship between M and Y, and thus false positives and negatives could occur. We also mention that more complicated relationships, such as the presence of multiple factors, will likely not be detected by our method.

2. The issue of the potential for multiple linked causal variants deserves particular attention in the Collaborative Cross, given the potential for large haplotype blocks with many tightly linked variants. It would be helpful to remind readers of the approximate haplotype size / rate of recombination in the Collaborative Cross, which determines the resolution at which it is possible to pinpoint causal genetic variants in this study. Then, this should be discussed and analyzed in more detail with respect to colocalizing eQTL and chromatin-QTLs. For any given pair of colocalizing eQTL and chromatin-QTLs, how many variants are in perfect LD? This affects our interpretation of how often the chromatin peak indeed causally mediates the effect on gene expression, versus how often the two effects might be caused by different tightly linked variants.

We agree this is an important point. In (Srivastava *et al.* 2017), they provide summaries of the number of haplotype blocks and their size distribution in both the most recent common ancestors (MRCAs) and sequencing CC strains:

In the CC mice, the median number of haplotype blocks is 121 (range 90-167) and the median haplotype block size is 16.3Mb (range 20Kb – 171Mb) in the autosomes. They report ~50 million variants across all CC strains, with 40 million identified with high confidence, resulting in ~16 variants/Kb. This implies that we would expect an average of 1,000 variants in each haplotype block, presumably in high LD, representing the potential for many variants within either the chromatin or expression QTL to segregate together. This underlies the challenge in determining in either QTL which is the casual variant, given that it is observed (genotyped), or if there is indeed a single causal variant. We do note that if there are multiple causal variants, the haplotype-based analysis does implicitly model this LD and can thus more powerfully detect QTL.

As indicated by Reviewer 3, it is possible that the casual variation is different between chromatin and expression, and this cannot be determined directly from the haplotype-based QTL analyses we performed, nor could it be determined from a more conventional SNP association analysis, both requiring follow-up experiments to confirm. Nevertheless, the haplotype-based analysis, given that it incorporates LD across multiple variants, contains more information about similarities across founder haplotype effect patterns, providing stronger support of related causality than SNP associations or effects. We have added this information to provide greater context for co-localizing QTLs to the “QTL detection: local (L), chromosome-wide (C), and genome-wide (G)” section of the Methods.

3. The initial step in the pairing procedure involves looking for signals located within 10 Mb of each other. However, the fact that there are eQTLs for the same gene (or cQTLs for the same peak) does not indicate that such QTLs could be caused by the same variant, and the authors follow up with mediation analysis. It would improve clarity to omit the results on page 5, lines 121-127; and instead only report the numbers in lines 130-133.

We understand that as presented, the clarity of these results could be improved. However, we would prefer that the numbers presented on lines 121-127 of the original manuscript should remain as we believe this information provides important context for the results in lines 130-133, which simply reports the number of correlated pairs. The initial numbers give a sense for how many total pairs were considered within each pairwise comparison of tissues. Nonetheless, consistent with Reviewer 2’s suggestion, we have combined the sections and edited them to improve clarity.

4. Additional evidence that their statistical approach for detecting mediation is well-calibrated (e.g., qq plots, power analysis, etc. based on real data and simulated data) would strengthen the paper.

Mediation analysis, particularly in the context of genomics, is a growing area of interest. Many past studies that deeply explored the fundamental methods and their statistical properties and challenges (Baron and Kenny 1986; MacKinnon *et al.* 2007; Imai *et al.* 2010) do not reflect the unique challenges and restrictions posed by genomics data. We agree with Reviewer 3 that more formal investigations of the statistical properties of mediation in a genomics setting could be valuable to the genetics field at-large; however, we posit that such a study is outside the scope of the work presented here. There are a number of significant challenges in performing such a study.

In terms of doing a simulation-based power analysis for mediation, the mediator relationship is far more complicated than the QTL relationship (Keele *et al.* 2019), due to the potential dynamics between M (mediator) and Y (gene expression), including reverse causality, feedback, variable measurement errors, presence of multiple mediators, and unobserved mediators in the presence of correlates, all of which would strongly influence sensitivity and specificity. Considering all these valid possibilities would require extensive simulations, as well as considerable effort regarding description and presentation. Further, there currently is not consensus in mediation procedures, which would also motivate evaluating multiple methods.

Towards evaluating the statistics from the actual data, the histograms for the mediation empirical *p*-values are in line with the FDR expectation (uniform distribution with a shelf representing enrichment in small *p*-values), though with a notable reduction in larger (less significant) *p*-values than expected in a traditional association context. We believe this reflects the fact that mediation is only tested at potential mediators for detected QTL. For instance, were all QTL truly mediated and we had infinite power, the expected mediation *p*-value distribution would be a point mass at 0, whereas that would not be the case for QTL analysis where there are many genes that do not possess a QTL. For this reason, we do not expect the conventional distribution metrics to apply to the mediation statistics. We do note that the mediation *p*-values, including insignificant ones, are now available in the raw results files in the figshare repository.

Despite the incomplete nature of the field’s understanding of mediation in the context of genomics, both in terms of its limitations and statistical properties, we see that it can provide strong functional candidates, particularly while considering additional information about the candidates, such as the zinc finger and chromatin mediators of *Akr1e1*. Towards this end, we have attempted to be conservative in declaring mediation through the use of empirically-derived *p*-values.

**Comments on power analysis:**

1. The value of this resource would be enhanced if the results of each variant-gene-tissue QTL test were reported (regardless of significance).

We appreciate the suggestion to include association statistics and effect size estimates for all markers/mediators as ideally this would provide additional context and potential use from our study. However, considering results would be required for every combination of tissue, conditional stage, experiment (gene expression or chromatin accessibility), and analysis (QTL or mediation analyses), it would represent an ambitious undertaking. Summary results of this kind are valuable for the large epidemiological studies used in human GWAS because the underlying data is not readily shareable and the summaries still allow for some degree of secondary analysis*,* *eg.* meta-analyses. For this study, however, we are able to make the underlying data publicly available (which we have stored at figshare), and the data can be reanalyzed from scratch or by running our software; for the latter, scan results, including effect sizes, can all be generated within a day, while permutation scans would require a few days to weeks, depending on the computational resources available.

Towards making the results of this study more easily available as a resource to the community, we have also included unprocessed QTL results tables in the figshare repository. These do not include every association and effect size for all loci and trait combinations, but does include the maximum association per trait, and thus includes potential QTL that did not meet the detection criteria, and could also be used to explore large-scale properties of the QTL and mediation *p*-values.

To allow all aspects of our results to be reproduced, we now provide example code using our miQTL R package to reproduce all the results as supplemental files (Files S26-S31) along with the normalized gene expression, chromatin accessibility matrices, and the CC founder haplotype mosaics for the analyzed strains at figshare (doi:10.6084/m9.figshare.9985514). Processed results files for all eQTL, cQTL, and mediations are provided as Files S8-S22.

2. Furthermore, the authors should calculate and report the power to detect effects of a given size for each variant-gene-tissue test (e.g., What is the power to detect 25% or 50% effect sizes? What is the smallest effect size that they are 80% powered to detect?). The authors report that, on average, the study has approximately 80% power to detect 55% effect sizes — but, with the data in hand, this can now be precisely calculated for every QTL test to account for the expression of the specific gene, etc. This would allow future users of this resource to understand whether, in cases where no eQTL is detected, what effect sizes the study was powered to detect.

We see the logic and potential value of this comment. However, conducting a reliable empirical power analysis based on these data would be problematic. Retrospective calculation of the effect sizes is in general problematic due to the Beavis effect, also known as the Winner’s curse, whereby the set of effect sizes estimated are on average inflated; based on work in (Keele *et al.* 2019) we would expect such inflation to be present in our data. This is also discussed in the last section of our Discussion.

A further complication is that power will vary with how strictly the false positives are controlled in a specific analysis. As such, technically, each type of our analyses (G, C, L genome-wide and L chromosome-wide) for this study would have different power. The reported 80% power to detect QTL with 55% effect size is based on a previous study in which we deeply characterized power in the CC, based on observed genomes, through the use of simulations (Keele *et al.* 2019), and which would best correspond in this paper to our Analysis L genome-wide. Our main objective in stating the expected power to detect 55% effect size QTL was to show that our observed effect sizes corresponded closely to simulated expectations, based on a broadly acceptable level of significance.

3. Information on power is furthermore very important for assessing whether a QTL is “tissue-specific”. If the test for a given variant-gene pair has substantially different power in two different tissues, this could lead to it being detected in one tissue but not the other for technical reasons. Was power considered in assessing tissue specificity?

On the subject of whether a QTL is tissue-specific, we are lenient when defining QTL pairs by allowing QTL from our stricter analyses to be paired with QTL only detected by our more lenient analyses, as exemplified by *Cox7c* (Fig3) in which a local-eQTL is most strongly detected in liver but highly consistent cross-tissue. This pattern is consistent with a multi-tissue QTL. Claiming a QTL is truly tissue-specific is challenging, and we did not mean to suggest that we were providing definitive evidence for this. Related to this point, we now refrain from using the term “tissue-specific” except in one section where we clarify that we mean specific across the three tested tissues and in the context of correlated haplotype effects. When QTL are detected in multiple tissues for the same gene or chromatin region but the haplotype effects are lowly or uncorrelated (or even anti-correlated), we believe this is greater evidence for some degree of tissue-specificity. Consider our example of *Pik3c2g*, where each tissue has local-eQTL, but with distinct effect patterns. Moreover, the eQTL in kidney and lung are highly significant, and each eQTL’s effect patterns are highly consistent with the mouse sub-species contributions to the founder haplotypes, localizing the eQTL in each tissue to different regions of the local region around *Pik3c2g*. Taken together, we believe this is strong evidence for the QTL acting very differently, and indeed likely represents effects of different variants. We agree, though, that it may be the case that some QTL are actually present in tissues, but below the detection threshold of our most lenient analysis (L chromosome-wide). But, this is different from detecting co-localized QTL with inconsistent haplotype effects, which is how we characterize tissue-specificity here.

4. **“These QTL mapping results are largely consistent with primarily Mendelian genetic regulation of molecular traits.”** This implication does not seem correct to me, as necessarily the analysis is biased towards finding large effects. The study is not powered to find small-effect, polygenic regulation of molecular traits. The results here do not broadly inform the genetic architecture of molecular traits, other than that, for some genes, large-effect associations do exist.

We agree with this point and concede that we over-stated that this study suggests molecular traits have simple genetic architecture. As Reviewer 3 states, we meant to suggest there are molecular traits with simple genetic architectures, and that for these traits, the CC can be used effectively for genetic mapping. This is certainly more difficult for physiological traits (*eg*. weight in humans) that are often more consistent with the infinitesimal model of quantitative genetics. We have added text to this effect in the last paragraph of the Discussion.

**Minor points:**

1. Fig. S21. Panels (A) and (B) are referred to in the caption but not labeled in the plot.

Correction made. We removed (A) and (B) references in the caption, and replaced them with [top left] and [bottom left] because detailed labeling of each subfigure was unnecessary for clarity.

2. Page 12, Line 365: **“our analysis has shown strong evidence that it acts causally by inducing a heterochromatic state near the Akr1e1 promoter.”** Accessible/non-accessible does not strictly correspond to euchromatin/heterochromatin. Furthermore, the evidence presented does not suggest, as the authors seem to be implying, that ZFP985 acts directly on the promoter of Akr1e1. I would suggest: “… that it leads to reduced accessibility at the Akr1e1 promoter”

We agree and have made the suggested edits.

**Have all data underlying the figures and results presented in the manuscript been provided?**
Large-scale datasets should be made available via a public repository as described in the *PLOS Genetics* [data availability policy](http://journals.plos.org/plosgenetics/s/data-availability), and numerical data that underlies graphs or summary statistics should be provided in spreadsheet form as supporting information.

Reviewer 1: Yes

Reviewer 2: None

Reviewer 3: No: Raw genotype and RNA/ATAC data are not yet available. Full summary statistics of association tests and mediation analysis are not available.

We have updated the supporting information for the work presented here. The processed data used to generate all these results, including gene expression, chromatin accessibility, CC strain haplotype mosaics, are all available now at figshare (doi:10.6084/m9.figshare.9985514). New supplemental R code files (Files S26-S31) have been added that can reproduce all results presented in text and figures in the manuscript and supplemental tables and figures.

The raw sequence data (RNA-seq and ATAC-seq) are currently awaiting approval by GEO, and will be available before publication. Notably, we did not generate raw genotype data for these mice, instead using haplotype mosaic information available from UNC (<http://csbio.unc.edu/CCstatus/index.py>) and SNP genotype data from ISVdb (<https://isvdb.unc.edu>), which were derived from different individuals of the same CC strains.

Baron R. M., and D. A. Kenny, 1986 The moderator–mediator variable distinction in social psychological research: Conceptual, strategic, and statistical considerations. J. Pers. Soc. Psychol. 51: 1173–1182. https://doi.org/10.1037/0022-3514.51.6.1173

Chick J. M., S. C. Munger, P. Simecek, E. L. Huttlin, K. Choi, *et al.*, 2016 Defining the consequences of genetic variation on a proteome-wide scale. Nature 534: 500–5. https://doi.org/10.1038/nature18270

Dudbridge F., and B. P. C. Koeleman, 2004 Efficient Computation of Significance Levels for Multiple Associations in Large Studies of Correlated Data, Including Genomewide Association Studies. Am. J. Hum. Genet. 75: 424–435. https://doi.org/10.1086/423738

Imai K., L. Keele, and T. Yamamoto, 2010 Identification, Inference and Sensitivity Analysis for Causal Mediation Effects. Stat. Sci. 25: 51–71. https://doi.org/10.1214/10-STS321

Keele G. R., W. L. Crouse, S. N. P. Kelada, and W. Valdar, 2019 Determinants of QTL Mapping Power in the Realized Collaborative Cross. G3 (Bethesda). 9: 459966. https://doi.org/10.1534/g3.119.400194

MacKinnon D. P., A. J. Fairchild, and M. S. Fritz, 2007 Mediation Analysis. Annu. Rev. Psychol. 58: 593–614. https://doi.org/10.1146/annurev.psych.58.110405.085542

Srivastava A., A. P. Morgan, M. L. Najarian, V. K. Sarsani, J. S. Sigmon, *et al.*, 2017 Genomes of the mouse collaborative cross. Genetics 206: 537–556. https://doi.org/10.1534/genetics.116.198838

Valdar W., J. Flint, and R. Mott, 2006 Simulating the collaborative cross: power of quantitative trait loci detection and mapping resolution in large sets of recombinant inbred strains of mice. Genetics 172: 1783–97. https://doi.org/10.1534/genetics.104.039313
